# Hydrogen mini-Factory for domestic purposes (wind version)

**DOI:** 10.1038/s41598-023-40205-6

**Published:** 2023-08-12

**Authors:** Dany Azig

**Affiliations:** Faculty of Environment, University of Tehran, Tehran, Iran

**Keywords:** Environmental sciences, Energy science and technology

## Abstract

The combination of wind energy as a source of production and hydrogen as a carrier and reservoir of energy has been a successful partnership. The unstable nature of wind and the long-term storage capability of hydrogen make them a prosperous pair. Many researches have been done in this field. In most of these researches, the focus has been on the production at the scale of wind farms or on the wind potential of the region. But in this project, the goal was to answer this question: is it possible to meet the energy needs of a household using the combination of wind energy and hydrogen? This project has created a step-by-step algorithm to answer this question. This algorithm starts by modeling the wind speed and finally ends by determining the overall dimensions of the system, including the active surface of the electrolyzer and the surface covered by the turbine. In this way, various components of the mini-Factory, such as electrolyzer, wind turbine, generator, and converter, have been investigated. Finally, an effort was made to select the most optimal operating conditions as well as the appropriate type for each of these components to achieve the expected output.

## Introduction

Water electrolysis is one of the cleanest methods in hydrogen production. This process is generally divided into three methods, Alkaline Electrolysis, Solid Oxide Electrolysis, and Proton Exchange Membrane (PEM) Electrolysis^1^. Each of these methods has its advantages and disadvantages, which must be chosen depending on different conditions. In this project, PEM Electrolysis was used to produce hydrogen. The reason for this is the superiority of this method to meet the conditions of this project. At first, the positive characteristics of PEM Electrolysis, including high efficiency, high current density, high purity of produced hydrogen, and higher hydrogen production rate, were the reasons for the choice. The other reason for selecting this method is its quick response^2^. Wind energy is constantly fluctuating over time; As a result, to avoid the loss of the generator's productive power and its momentary conversion to hydrogen, a quick method should be coupled with the wind system. On the other hand, the low operating temperature eliminates the need for a high-temperature heat source, which is also important in instant production.

Wind energy production systems cover a wide range. But generally, systems based on wind turbines have established their place in this field for years. Wind turbines have different categories, and one of these categories is the turbine axis. Based on the orientation of the turbine rotor axis, they are divided into two categories: horizontal axis and vertical axis. Both of these categories, from small-scale to large-scale turbines, are produced and supplied both at the experimental level and at the industrial level^3^. The choice of each of these two types should be based on different conditions. In general, the advantages of horizontal axis wind turbines (HAWT) are higher efficiency and more power generation^4^; But on the other hand, their implementation and operational costs increase due to their greater complexity. As a result, vertical axis wind turbines (VAWT) are usually used for domestic scales. Among their advantages are the need for a smaller foundation, lower construction costs, easier maintenance due to the location of the turbine generator on the ground, and less noise. This makes them a more suitable choice for urban environments and close to residential homes^5^.

In this project, the goal is to produce the energy needs of a household. Space limitations and, as a result, the closeness of the turbine to the home environment caused the vertical axis turbine to be considered in the design.

As mentioned, wind energy is an unstable energy and its production must happen at the moment; For this purpose, for household scales, which are not connected to the grid, an energy storage system is one of the basic pillars of the system. There are different ways to store wind energy, including batteries, current batteries, flywheels, etc.^6^; But hydrogen, as a versatile energy carrier, was chosen to meet the household's need for electricity, gas, and car fuel. In this way, by storing wind energy in the form of hydrogen, the shortcomings of both systems are largely eliminated and the energy needs of a household are met.

Incorporating the two ideas of wind energy and water electrolysis is an attractive idea that has led researchers to innovate for years. Focusing on wind energy potential measurement, Alavi et al.^7^ have used two approaches. The first approach is to use the wind data itself, and the other approach is the Weibull distribution function. They investigated four different VAWT. They have also used alkaline electrolysis and a linear model between wind power and hydrogen production. Focusing on the wind sector and examining the economic aspects, Rezaei et al.^8^ investigated the levelized cost of electricity (LCOE), the energy efficiency of hydrogen production, the levelized cost of hydrogen (LCOH), and the payback period. They considered three scenarios with five degradation rates for these four mentioned parameters. In a similar work, Almutairi et al.^9^ performed a technical, economic, and carbon footprint assessment for hydrogen production from wind energy. In the technical evaluation, they used the data of four stations, at three heights and the Weibull distribution function. They have included the estimation of the energy pattern factor, the probability of wind speed greater than 5 m/s, wind power density, annual power output, and annual hydrogen output in their technical evaluation. LCOE, LCOH, and payback period were the parameters they used in the economic evaluation. The evaluation of the consequent CO_2_ reduction in the two scenarios was the other aspect of their work. They have also used a linear relationship to convert wind power into hydrogen production, as in the previous two studies. From the point of view of wind energy, Javaid et al.^10^ have used the data of wind speed, direction, and gust to predict with the help of statistical and machine learning models. They have used three models of linear regression, support vector regression, and long short-term memory to predict the characteristics of the wind and the amount of hydrogen produced.

Meier^11^ has investigated the potential of hydrogen production in an offshore wind farm. In his work, he has used real data obtained from other farms. In his modeling, he used PEM and solid oxide electrolyzers. Also, it has investigated the economic and investment aspects of hydrogen production, based on the price of fuel. In another paper on the field of wind farms, Sarrias-Mena et al.^12^ coupled a PEM electrolyzer with their system with the aim of preventing the waste of excess load generated in a wind farm. They modeled all components of the wind system, including turbines, generators, controllers, and converters. Also, four different models were used to model the PEM electrolyzer section, to check the difference in the results of each model. In a similar work, Wilberforce et al.^13^ investigated the overall hydrogen production rate for an integrated system of vertical axis wind turbine and PEM electrolyzer with the help of mathematical models, supported by real data. They modeled hourly wind data for a year. They also investigated the correlation between the real data and the mathematical model for the developed electrolyzer model.

In this project, the approach is slightly different from the mentioned articles. Imagine that a household needs a certain daily amount of energy. What conditions must be established in order to provide this amount of energy? Basically, looking backward has been considered in the design of this project. That means the final goal is the amount of energy production, with a certain amount; Therefore, based on that, the required amount of hydrogen, the dimensions of the electrolyzer, the dimensions of the wind turbine, and finally the profile of the wind speed have been determined. This article has formulated a series of concepts to address the question, “Can we take a reliable look at wind energy?”.

In the first step, the question has been answered, what wind profile should I have so that I can receive this amount of hydrogen or energy per day? This question has been investigated by determining a wind profile using random numbers with gamma distribution.

In the next step, the type of wind source was investigated and solutions were presented to extract energy from each of these sources.

On the next stage, the components of the wind sector, including the type of turbine and generator, were examined to select the most appropriate type for this project.

In the next phase, the working conditions of the PEM electrolyzer and their optimal values were investigated. Also, a solution was presented to increase the lifetime of cells.

Finally, a working algorithm was developed to calculate the step-by-step Hydrogen mini-Factory design. This algorithm is completely open and can be developed for more complex conditions. The code of this algorithm is written in Python programming language, which makes it easier to develop.

## Hydrogen mini-Factory

Solar and wind energy are two renewable energies that can be found in most parts of the world. Their intensity and strength depend on various factors. In natural environments outside the residential area, usually due to the lack of urban pollution and tall buildings, solar energy can be easily obtained. But on the other hand, wind energy is somewhat unpredictable. So today, even around the freeways, a kind of strong and efficient wind flow can be found^14^. Also, tall buildings typically create wind channels that blow around them^15^. As a result, wind can be found, unlike solar radiation, even in dense urban environments. For this reason, this unique feature of wind has made it a resource available to everyone. Also, some areas of the world, from the Nashtifan in Iran to Scotland in the United Kingdom, have strong and permanent wind currents, which is a definite source of energy production.

The general conditions to produce hydrogen assumed that there is no access to clean water. As a result, only with the help of wind energy, distilled water must be provided to produce hydrogen, and then hydrogen is generated by the PEM electrolyzer.

To find the amount of wind power at a point on the earth, there are different solutions, the most accurate of which is to measure the speed and direction of the wind with the help of anemometers^16^. The hidden goal of the project is to create a production plant anywhere in the world; As a result, a general process has been designed to calculate the overall dimensions of the system, so that it can be customized for each specific case, depending on the conditions.

### Simulation of wind speed

The first step in determining the amount of wind power is to determine its speed. Wind speed depends on many variables, including height from the ground, surface coverage or roughness, time of day, month or even year, etc.^17^. One of the main challenges of this project is to determine the wind speed profile so that it can be considered a good representative of real conditions. But the constant variation of wind speed, how should it be modeled?

Random phenomena can be well modeled with the help of statistical distributions. Typically, in the case of wind, the Weibull distribution is used^18,19^; However, the gamma distribution function also seems to be an interesting option. But why is the gamma distribution function suitable for wind modeling?

**Figure 1:**
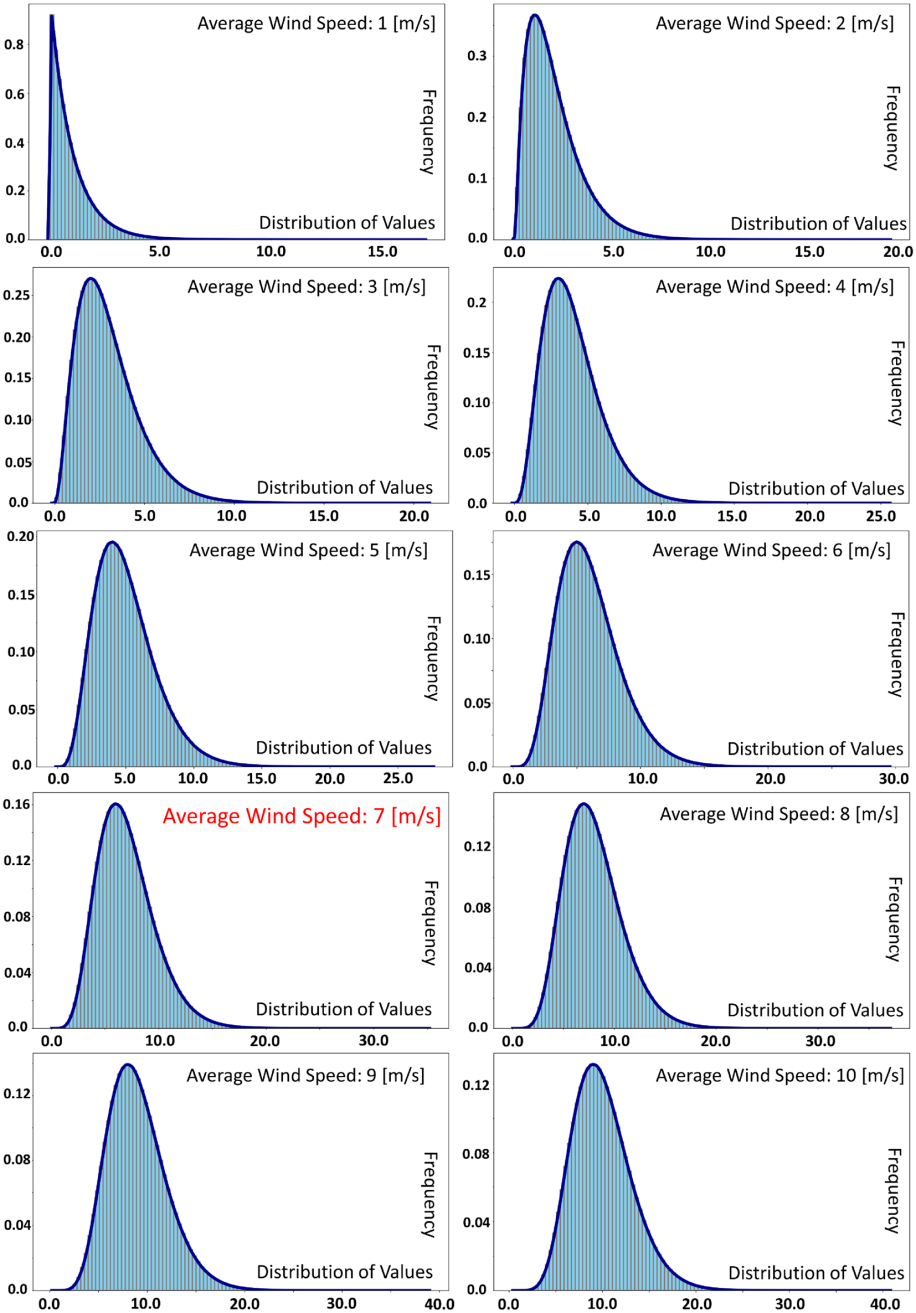
Histogram and density of the gamma distribution function, for 10 random datasets created and used in the calculations of this project, from the average wind speed of 1 m/s to 10 m/s.

The gamma distribution function has two important properties that make it a good choice for constructing a random data set as a representative of wind speed. The first attribute is that this function can be controlled with only one parameter, which is called the shape parameter^20^. This shape parameter is essentially the mean of the data, which, as a result, is a great help in determining wind speed. This feature is a very useful option, because to determine the value of the average wind speed at a point, by setting the shape parameter equal to that value, the distribution around that number is easily obtained. Another feature is that this function has a skewness to the right for small and close to zero values of the shape parameter. As the value of the shape parameter increases, this function becomes similar to the normal distribution function and the skewness disappears^20^. This feature is very similar to nature. Take a look at Fig. 1 and see the changes in the shape of distributions with means from 1 to 10. At low wind speeds, the intensity of the wind may increase at a time of the day or year, which is usually happened in spring. That is, even in low averages, a high amount of wind may occur, which causes a right skewness. But in windier places, there are always times when the wind subsides, so, in addition to windy periods, there are also periods of low wind, therefore a normal distribution is a more suitable representative for those conditions. As a result, gamma distribution was chosen for this project.

Wind speed generally creates two scenarios for mini-Factory design. Points with an average speed above 4 m/s and below this value. Why is this? Because wind turbines have a minimum speed called cut-in speed, which is the amount of current that must be maintained for the turbine to start rotating. The cut-in speed is different for any turbine, but 4 m/s is common for VAWTs^21^.

What should the areas with lower speeds do? According to the wind speed gradient, the relationship between wind speed and height is a power relationship, which is described completely in the “Methods” section. This relationship states that the higher the altitude, the higher the wind speed. Of course, the intensity of these changes depends on the effects of the earth's surface^22^. Therefore, for points where the average speed is less than 4 m/s, the option of increasing the height is a suitable solution.

### Wind power generation system

In this section, wind energy conversion systems for different conditions are discussed. In the beginning, the first scenario mentioned at the end of the previous section will be examined. What should areas with wind less than 4 m/s do? In these areas, if there are no obstacles, it is possible to increase the height to reach higher wind speeds. In these cases, one of the most common tools for generating wind power is Airborne wind energy (AWE) systems. These systems are in the form of a balloon or kite, sent into the height, and connected to the ground with a cable. These systems have advantages such as no need for foundations and quick setup^23^.

The second scenario is for areas where the average wind speed is more than 4 m/s. Here, the wind source itself is a factor in creating two different scenarios. In the first case, wind sources are considered, which is caused by an anthropogenic factor. For example, wind sources around highways, which are created by the movement of automobiles. Or in another example, wind currents form wind tunnels around tall buildings. In this case, usually, a small turbine farm, on the side of the highway^24^, or a number of turbines in a cumulative manner, in front of a building^25^, may be the most suitable solution. For this purpose, the detailed investigation of wind distribution and its speed profile is very important because these sources usually have a constant direction and dimensions.

The last scenario is the main goal of this project. Areas with favorable wind speeds that originate from a natural source and as a result have good cohesion and continuity. In this situation, a medium-scale household turbine can be considered for energy production. Specifically, a straight-bladed Darrieus VAWT was chosen as the ultimate goal due to its simplicity in design and suitability for residential environments. In the mini-Factory design process, due to the complexities of wind speed and wind turbine efficiency, only the rated efficiency of VAWTs, which is around 40%, was used^25^.

Based on the surface and efficiency of the turbine, as well as the wind speed profiles, the output power of the turbine can be estimated for any condition. As a result, by estimating the output power of the wind turbine, it is possible to evaluate the amount of hydrogen production with the help of a PEM electrolyzer.

After choosing the type of wind turbine, the electrical power conversion system must be considered. Since PEM electrolyzers work with Direct Current, as a result, this power conversion system should preferably have a DC output. Different types of generators are used for wind turbines, which include: permanent magnet DC generators, permanent magnet synchronous generators, wound rotor synchronous generators, squirrel cage induction generators, doubly fed induction generators, etc. Usually, based on the type of fixed or variable speed, any of these generators are used to produce electricity. DC machines are usually used for small power. They are expensive to maintain and repair because of the parts they use, like brushes^26^. But for this project, they are a good choice because they don't need an AC-DC converter.

Another option that can be used is permanent magnet synchronous generators (PMSG). These types of generators have many advantages, including variable speed power generation that increases efficiency and production of power. Another important feature of them is converting the generated power from AC to DC and then DC to AC for transmission to the grid^27^. This creates a suitable situation. Because, after converting the power from AC to DC, it is possible to transfer the generated electricity to the electrolyzer without needing to convert again. Also, the wide range of different arrangements for the machine side converters, makes it possible to find the most suitable option that is compatible with the electrolyzers.

### PEM electrolyzers, a good partner for the dynamic systems

PEM electrolysis is one of the most appropriate options for dynamic systems such as wind systems; Because PEM electrolyzers can maintain their performance even at high current density^28^. The efficiency of the electrolyzer depends on the current density, and the efficiency decreases with its increase^29^. Also, the overpotential of the concentration becomes significant as the current density increases. So, a ceiling should be set for the current density. This threshold is considered for the current density in some sources of about 20,000 A/m^2^^30^. This value was considered as a limit for determining the dimensions of the active surface of the electrolyzer during the design of the mini-Factory.

Other operating conditions are working temperature and pressure. For the mini-Factory, the temperature and pressure were assumed to be 80 °C and 100 kPa respectively. This temperature is the highest typical operating temperature for PEM electrolyzers. The reason for this choice is to reduce the amount of power required because as the temperature increases, the power required for production decreases. In the case of PEM electrolysis, another thing to consider is the useful life of the membrane, which depends on the temperature. As the temperature increases, the useful life of the membrane decreases^31^, so how should we deal with this problem? How to reduce the power requirement, while preserving the useful life of the system?

The solution that this project offers is that, since the current density is directly dependent on the input power of the wind and subsequently on the wind speed; Because of that, the current density is constantly changing with the wind speed. This is a great advantage because by controlling this current and combining it with electrolyzer cells, a dynamic system can be created. The calculations of this project determined 36 cells of 25 × 25 cm to produce 5 kg of hydrogen per day. The maximum power input to the electrolyzer is around 92 kW. This power is changing at every moment with the speed of the wind. It is enough to apply this power to several cells in a stepwise manner by increasing a threshold value in each step. For example, the power below 23 kW should be applied to the first 9 cells, 46 kW to the first 18 cells, 69 kW to the first 27 cells, and finally, the maximum power should be applied to all the cells. This causes some cells to be at rest when the power is not maximum. In this way, resting cells approach the end of their life later, in other words, the useful life of the entire electrolyzer increases.

### Analysis of outputs

**Figure 2:**
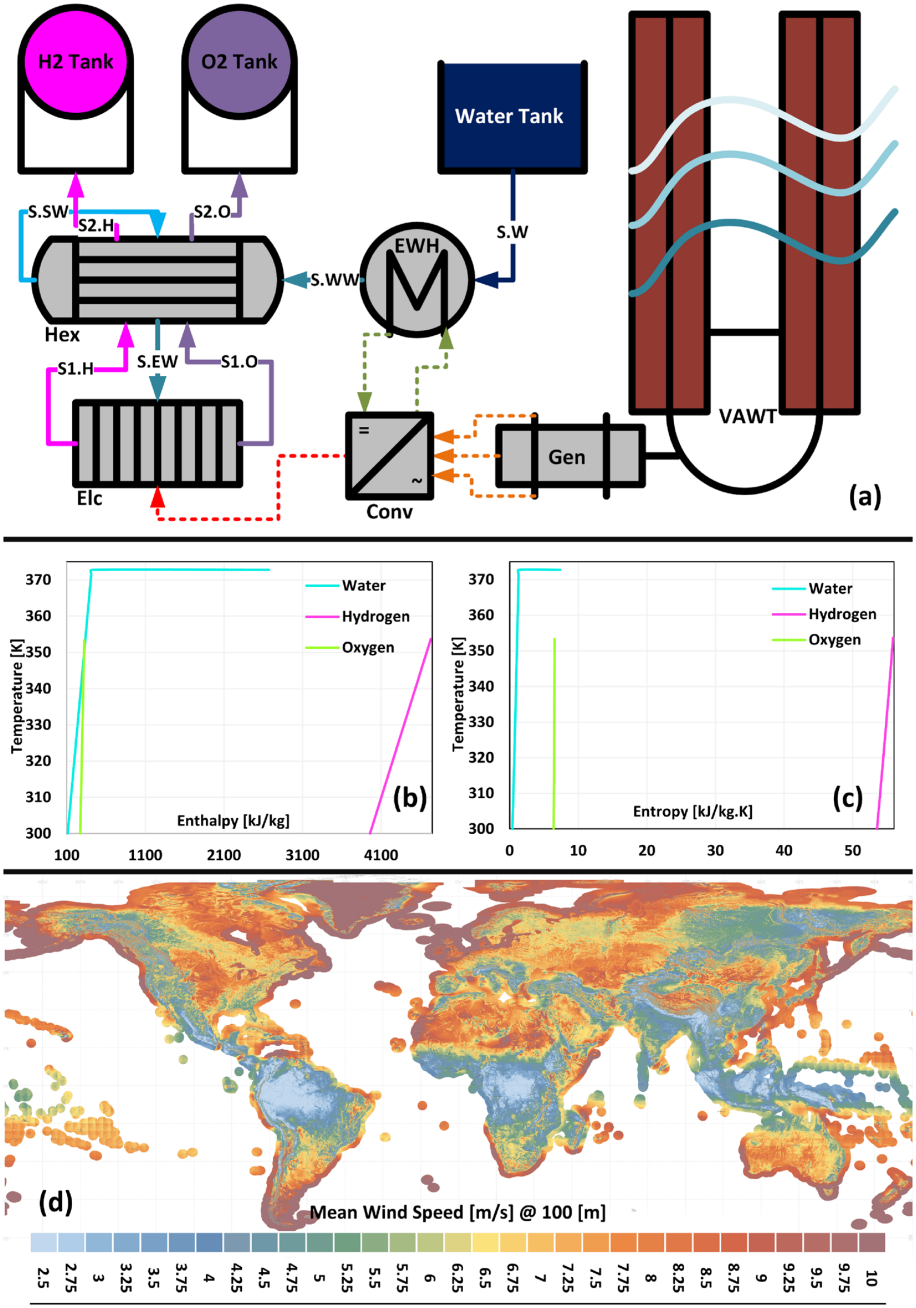
(**a**) Flowchart of the components of the mini-Factory. (**b**) Enthalpy diagrams of materials participating in the water electrolysis reaction. (**c**) Entropy diagrams of materials participating in the water electrolysis reaction. (**d**) Global map of the average wind speed at a height of 100 m, published by the World Bank Group on the website (https://globalwindatlas.info/en/download/high-resolution-maps/World).

In Fig. 2a, the Hydrogen mini-Factory components are depicted. The parameters used for the design of each of these components are given in Table 1. On the other hand, the operating conditions and thermodynamic properties of each stream in this figure are given in Table 2. These features include temperature, pressure, and mass flowrate that selected or calculated for these streams. Also, the specific thermodynamic properties of these streams, including enthalpy, entropy, and specific exergy, have been determined. With the help of these features and the equations mentioned in the “Methods” section, the balances of mass, energy, entropy, and exergy of each component have been calculated. Table 3 shows the output of these four balances. Let's take a look at Table 3 to see which of the components of the mini-Factory had the most losses.

**Table 1:** Defined and calculated parameters for Hydrogen mini-Factory.

Parameter	Value	Unit	Description	References
Hydrogen mini-Factory				
Goal				
Hydrogen mass flow rate	5	kg/day	Daily production of 5 kg of hydrogen by mini-Factory (equivalent to 1825 kg per year)	–
Energy and exergy efficiencies				
Energy*	28	%	Overall mini-Factory efficiency	–
Exergy*	27	%	Overall mini-Factory efficiency	–
Electrolyzer (defined parameters)				
Constants				
Gas constant	8.3144	J/mol K	–	https://www.nist.gov/
Faraday constant	96,485	C/mol	–	https://www.nist.gov/
Molecular masses				
Water	18.01528	g/mol	–	https://www.nist.gov/
Hydrogen	2.016	g/mol	–	https://www.nist.gov/
Oxygen	31.999	g/mol	–	https://www.nist.gov/
Operational conditions				
Cell temperature	353	K	–	^38^
Cell pressure	100	kPa	–	^38^
Structural parameters				
Electrolyte thickness	50 * 10^–6^	m	Nafion	^38^
Water content	10	–	For cathode (λ_c_)	^38^
Water content	14	–	For anode (λ_a_)	^38^
Charge transfer coefficients	0.5	–	For cathode (α_c_)	^30^
Charge transfer coefficients	0.5	–	For anode (α_a_)	^30^
Current parameters				
Exchange current density	10	A/m^2^	For cathode	^30^
Exchange current density	1.0 * 10^–5^	A/m^2^	For anode	^30^
Stack parameters				
Cell area	0.0625	m^2^	25 cm * 25 cm	–
Ambient conditions				
Temperature	300	K	–	–
Pressure	100	kPa	–	–
Electric water heater				
Efficiency	95	%	–	–
Energy and exergy efficiencies				
LHV for hydrogen	119,930	kJ/kg	At standard conditions: T = 25 °C and P = 1 atm	https://webbook.nist.gov/
Chemical exergy for hydrogen	116,760	kJ/kg	At standard conditions: T = 298 K and P = 1.013 bar	^50^
Electrolyzer (calculated parameters)				
Potentials				
Standard potential	1.183	V	At operational conditions	–
Reversible potential	1.183	V	In the determined molar composition	–
Actual potential*	1.928	V	–	–
Overpotentials				
Ohmic*	0.0064	V	For electrolyte	–
Activation*	0.159	V	For cathode	–
Activation*	0.58	V	For anode	–
Mass transfer rates				
Water**	0.0128	mol/s m^2^	Consumption rate	-
Hydrogen**	0.0128	mol/s m^2^	Production rate	–
Oxygen**	0.0064	mol/s m^2^	Production rate	–
Final parameters (required)				
Operating current density*	3075.859	A/m^2^	–	–
Hydrogen molar flow rate**	0.0287	mol/s	Equivalent to 5 kg/day	–
Electrolyzer surface	2.25	m^2^	–	–
Power density^**^	4.901	kW/m^2^	–	–
Heat density^**^	− 1.098	kW/m^2^	–	–
Entropy generation*	0.4075	kJ/mol K	Based on moles of hydrogen produced	–
Stack parameters				
Number of cells	36	–	–	–
Number of stacks	1	–	Series arrangement	–
Efficiencies				
Energy*	57	%	At the start of process	–
Energy*	65	%	steady-state	–
Exergy*	55	%	At the start of process	–
Exergy*	63	%	steady-state	–
Wind system (defined parameters)				
Gamma distribution function				
loc	0	–	The shift parameter to shift the distribution on the x-axis (0 equals no shift)	Given in the Statistical aspects of design section
scale	1	–	Scale parameter for scaling the distribution to the desired size (1 equivalent without changing the scale)	Given in the statistical aspects of design section
size	25,246,080	–	Equivalent to 80% of the number of seconds during a year	Given in the statistical aspects of design section
random_state	1	–	To store the generated random dataset in memory for consistency in results	Given in the statistical aspects of design section
Air density				
Molecular mass	28.965	g/mol	–	https://en.wikipedia.org/wiki/Density_of_air
Wind turbine specifications				
Efficiency	40	%	Rotor section	^25^
Efficiency	90	%	Electric section	–
H/R	2.8	–	Height to radius ratio	^42^
Triple speeds				
Cut-in	4	m/s	–	^21^
Cut-out	25	m/s	–	^21^
Rated	15	m/s	–	^21^
Wind gradient				
Hellmann exponent	0.34	–	For neutral air above human inhabited areas	^44^
Target speed	7	m/s	Reference height at 10 m	–
Wind system (calculated parameters)				
Gamma distribution function				
Shape parameter	7	–	Equivalent to the average wind speed of 7 m/s (selected speed)	–
Air density				
Density	1.161	kg/m^3^	Calculated in the environmental conditions specified for the electrolyzer section	–
Wind turbine specifications				
Overall efficiency	36	%	–	–
Required surface*	126.648	m^2^	–	–
Turbine height	13.316	m	–	–
Turbine radius	4.756	m	–	–
Rated power*	89.345	kW	The maximum output power of the turbine, equivalent to the rated wind speed (15 m/s), is approximately 90 kW	–
Wind gradient				
Equivalent height	3059.021	m	For an average wind speed of 1 m/s	–
Equivalent height	398.291	m	For an average wind speed of 2 m/s	–
Equivalent height	120.861	m	For an average wind speed of 3 m/s	–
Equivalent height	51.858	m	For an average wind speed of 4 m/s	–
Equivalent height	26.902	m	For an average wind speed of 5 m/s	–
Equivalent height	15.736	m	For an average wind speed of 6 m/s	–

*The calculation of these numbers was done by averaging over the number of dataset members (for more details, see “Statistical aspects of design” section).

**The calculation of these numbers by the method of summing all the values divided by the time period of 1 year (for more details, see “Statistical aspects of design” section).

**Table 2:** Operating conditions and thermodynamic properties of Hydrogen mini-Factory streams.

Stream	Temperature (K)	Pressure (kPa)	Mass flowrate* (kg/s)	Specific enthalpy (kJ/kg)	Specific entropy (kJ/kg.K)	Specific exergy (kJ/kg)
S.W	300	100	0.00052	112.65	0.3931	0
S.WW	322.2	100	0.00052	206.24	0.692	3.909
S.SW	372.76, x = 1	100	0.00052	2674.9	7.3588	472.528
S.EW	353	100	0.00052	335.3	1.07	19.568
S1.H	353	100	0.0000578	4722	55.86	61.4
S2.H	300	100	0.0000578	3958.3	53.519	0
S1.O	353	100	0.00046	321.8	6.567	3.88
S2.O	300	100	0.00046	272.71	6.4163	0

*Average values are calculated by dividing the total produced mass by the total seconds of a year (for more details, see "Statistical aspects of design" section.)

**Table 3:** Calculated thermodynamic parameters for Hydrogen mini-Factory components.

Component	Heat transfer rate (kW)	Work transfer rate (kW)	Entropy rate duo to heat (kW/K)	Entropy generation rate (kW/K)	Exergy rate duo to heat (kW)	Exergy destruction rate (kW)
Elc	− 2.471	11.028	− 0.007	0.0127	-0.371	3.807
Hex	–	–	–	0.0038	–	1.144
EWH	–	0.056	–	–	–	–
VAWT	–	11.084	–	–	–	–

The calculation of these numbers by the method of summing all the values divided by the time period of 1 year (for more details, see "Statistical aspects of design" section.)

**Figure 3:**
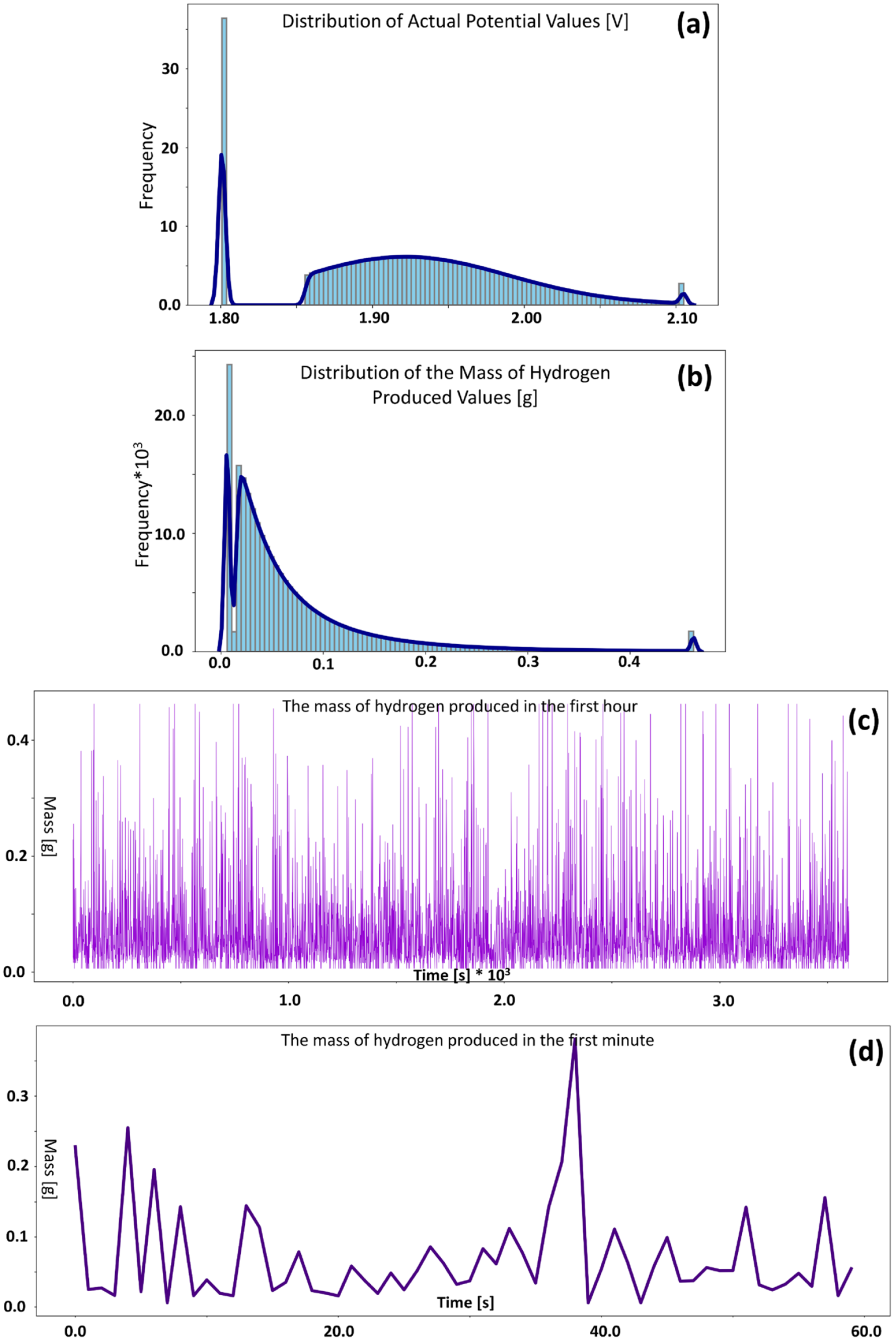
Histogram and density of the gamma distribution function, for (**a**) actual potential (**b**) mass of produced hydrogen; Graphs related to the dataset of the mass of produced hydrogen for time intervals (**c**) the first hour (**d**) the first minute.

The highest exergy destruction rate is related to the electrolyzer. Why did this happen? If we look at Eqs. (22) and (27), we will find that the exergy destruction rate directly depends on the entropy generation rate, and the entropy generation rate directly depends on the sum of overpotentials. Figure 3a shows the data distribution of the actual potential for the simulated wind data used in this project. A close look at this graph will clarify the reason for this high exergy destruction rate. In Table 1, the reversible potential of the cell is given, which is calculated to be 1.183 V. But according to Fig. 3a, the lowest value of the actual potential of the cell is 1.8 V, which means that the minimum amount of overpotentials is about 0.6 V or 1/3 of the total potential. It is a high value, which gradually increases with the increase of current density. The most important thing to note is that the distribution of wind speed and other variables, such as the mass of hydrogen produced (shown in Fig. 3b), exhibits a pronounced pattern, and the data is centered around a particular value. But on the other hand, the distribution of the actual potential of the cell, except for the initial value of 1.8, has an almost uniform distribution in the rest of the values. This shows that the change in the wind intensity and consequently the intensity of the current density doesn't change the intensity of the actual potential; As a result, this element cannot be easily controlled by controlling another element of the system. According to the equations mentioned in the “Methods” section, this element can only be controlled by operating conditions such as temperature and pressure, and the structural characteristics of the electrolyzer. On the other hand, in this project, an attempt was made to select the most optimal operational conditions to overcome these problems; However, again, the exergy destruction resulting from these overpotentials is significant.

In terms of energy, the wind turbine has the highest amount of losses. Because the wind energy entered in the space covered by the turbine is around 30 kW while the output power from the turbine is 11 kW as shown in Table 3. This issue has happened while, in this project, an effort has been made to choose the most optimal conditions in order to obtain the appropriate output. This shows that the limitations of wind turbines for power conversion are undeniable. According to the experience of this project, reducing the cut-in speed and increasing the rated speed will improve these conditions. Of course, methods to improve the performance of VAWTs are expanding day by day.

**Figure 4:**
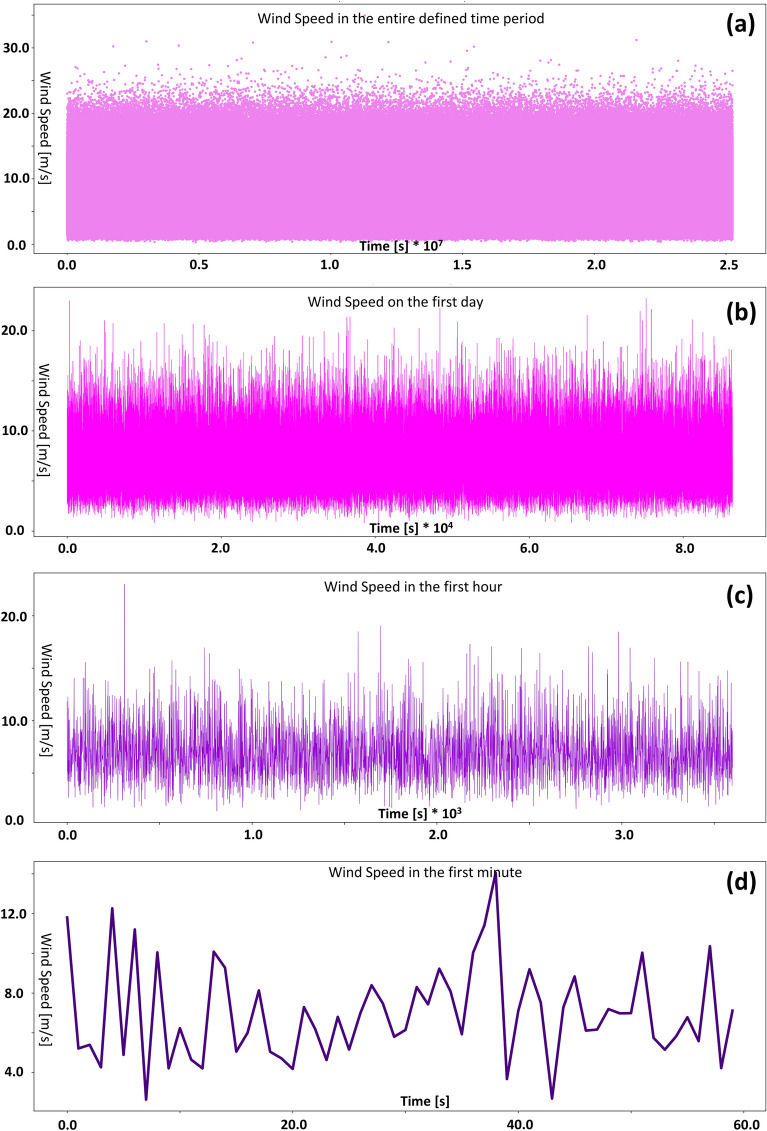
Graphs related to the dataset of the average wind speed of 7 m/s for time intervals (**a**) the entire dataset (80% of the year) (**b**) the first day (**c**) the first hour (**d**) the first minute.

Figure 1 shows the distribution diagrams of the constructed dataset for wind speed simulation. These graphs are gamma distribution with average natural numbers from 1 to 10. The number of members of each of these datasets is equal to 25,246,080. Among these 10 datasets, the dataset with an average of 7 was selected for the mini-Factory design. The selection method and the reason for the number of dataset members are given in the "The mini-Factory design algorithm" section. If these datasets are plotted as consecutive numbers, like the seconds of a year, in a graph, Fig. 4a is obtained. The high density of numbers has reduced the ability to interpret this figure. Now, for better representation, the first 86,400, 3600, and 60 numbers of this sequence were cropped and shown as representatives of the day, hour, and minute respectively. These three trimmed sets are shown in Fig. 4b–d. As can be seen from the images, the frequency of the data is extremely high, which is consistent with the nature of random numbers. But in reality, the wind doesn't fluctuate that much every second. That is why, for the modeling, the period of the whole year was assumed, so that, in a way, by putting all these data together, the final target value, which is the annual production of 1825 kg, is obtained. Equivalently, the hydrogen production load profile is shown in Fig. 3c and d for the first hour and minute of the dataset. However, the sum of these numbers doesn't necessarily equal 5 kg in 1 day, but the sum of these numbers in 1 year is 1825 kg.

## Discussion

Before going into the details of this project, let's take a look at the concept of on-site energy production and consumption. First, let's examine its disadvantages. The first point is to check the safety status of this system. The low operating pressure and temperature selected for the electrolyzer reduce the risks associated with the electrolyzer section to some extent; But hydrogen and oxygen produced gases can explode if not kept in proper conditions. Another problem is the large space occupied by the wind turbine. Choosing a vertical axis wind turbine has overcome this problem comparatively, but again, for places with high density, the need to increase the height of the hub is felt. Another point is the discussion of costs. The cost of this set, assuming the simplest equipment used, is calculated at around $200,000. In contrast, with the daily production of 5 kg of hydrogen, it takes 22 years to pay back this cost. But on the other hand, what are the benefits of this concept included in this project? The first and most positive point is not having to pay energy costs. As long as this system is active, it solves the need for electricity, gas, and car fuel. Simpler equipment reduces the overall complexity of the system, thus reducing maintenance costs. On the other hand, simple system checks can be left to the household itself. This idea makes the household more responsible for its energy use.

Did this project answer the question raised? If the conditions of this project are met, the answer is yes. Initially, the wind speed distribution should be a gamma distribution with a mean of 7 m/s for 80% of the year. The next issue is the type of system and operating conditions selected. The vertical axis wind turbine with the determined dimensions, the determined active surface for the PEM electrolyzer, and the considered energy conversion efficiencies and temperature and pressure should be similar to this project. If these conditions are met, it can be said that the combination of wind and hydrogen, wherever you are in the world, will make you unnecessary of energy expenses. But, what are the ways to develop this plan? The created algorithm can be widely developed. For example, in the part of wind turbine modeling, more accurate models can be used. Variable temperature and pressure can be used for electrolyzer operating conditions. It can be upgraded for multi-generation systems. Finally, it can be said that this project has created a strong computing base that can be used to determine the dimensions of a domestic system for energy production and storage.

## Methods

In this section, all the stages of the mini-Factory design are explained in detail. First, it should be mentioned that all the calculations of this project were done with the help of Python programming language. The written script is available in the Data Availability section. In this process, the math built-in module, as well as the libraries matplotlib version 3.5.2, numpy version 1.21.5, pandas version 1.4.3, scipy version 1.7.3 and seaborn version 0.11.2 were used. Also, Spyder version 5.2.2, an open-source cross-platform integrated development environment for scientific programming, was used to implement the Python programming language.

All the thermodynamic data used, for the materials participating in the mini-Factory design, were taken from the National Institute of Standards and Technology (NIST) website (https://webbook.nist.gov/chemistry/fluid/), which has made the relevant information freely available to the public. These thermodynamic data are depicted in Fig. 2b and c.

All defined and calculated parameters for the mini-Factory are also given in Tables 1, 2, and 3.

### The mini-Factory design algorithm

**Figure 5:**
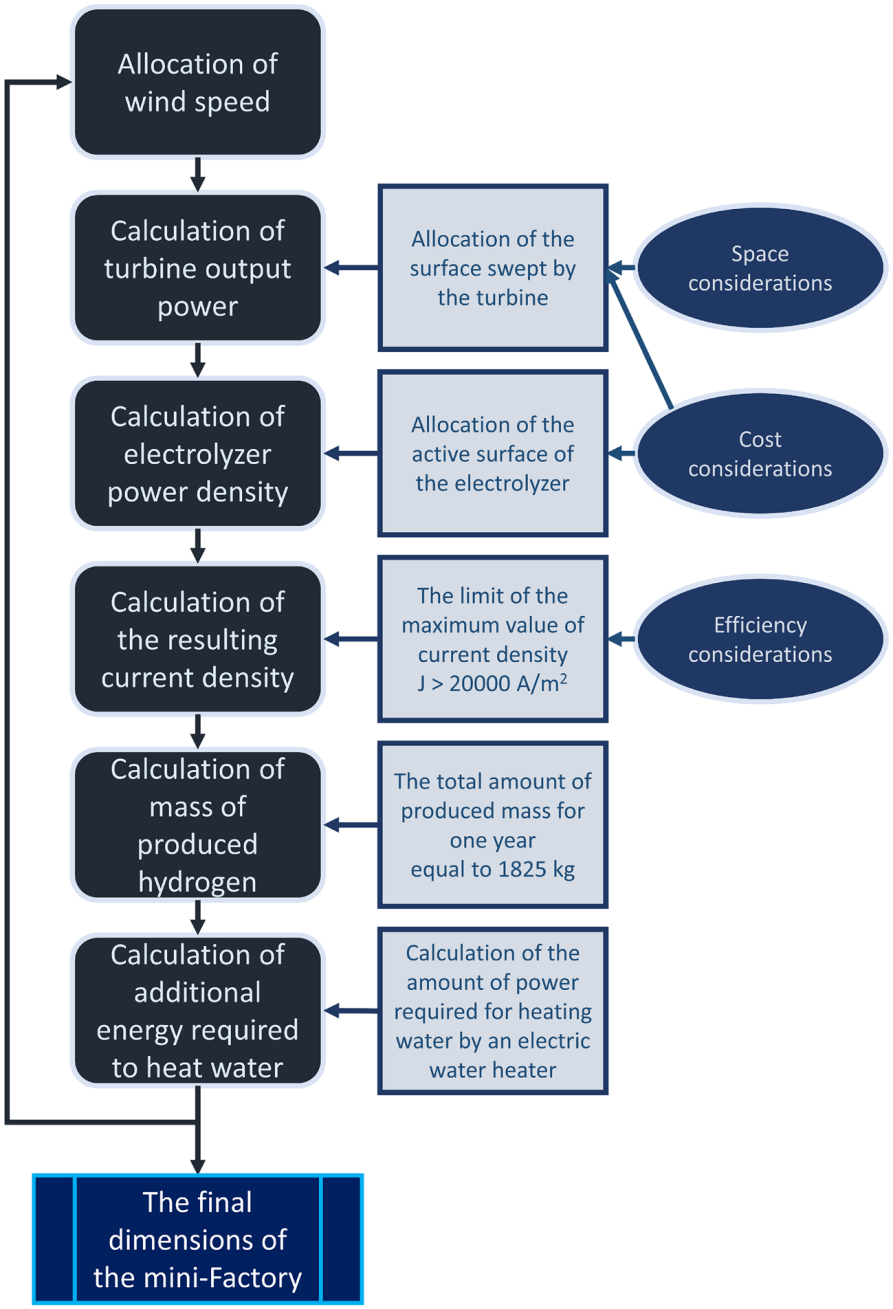
Algorithm of the design steps of the Hydrogen mini-Factory and finding the dimensions of its components.

The mini-Factory design process is summarized in Fig. 5. Each of these steps is explained below.

#### Allocation of wind speed

In the first step, determining the wind speed is the beginning of the design cycle. As mentioned, the gamma distribution function is the solution of this project to create a set of random numbers around a specific value. In general, the gamma distribution function is as follows (https://docs.scipy.org/doc/scipy/reference/generated/scipy.stats.gamma.html):

1 $$\mathrm{f}\left(\mathrm{x},\mathrm{ a}\right)= \frac{{\mathrm{x}}^{\mathrm{a}-1}{\mathrm{e}}^{-\mathrm{x}}}{\Gamma (\mathrm{a})}$$

Equation (1) is the gamma probability density function. With the help of this function, a probability value (usually between zero and one) can be attributed to a real number. Now, how can this function help us to generate wind speed? Python programming language is a powerful language that has many packages and libraries in the field of statistics. One of these libraries, which of course has many other applications, is the scipy Library. With the help of the stats module, from this library, you can create a set of random real numbers, which have a special statistical distribution. In this project, a dataset with gamma distribution was created around the natural numbers 1 to 10 with the help of the shape parameter explained in the Simulation of wind speed section, as shown in Fig. 1.

This random dataset around natural numbers from 1 to 10 can be equivalent to a set of wind speeds measured in an area. That means, during a certain period of time, a certain amount of data for wind speed is measured. To simulate these measurements, this random set is a suitable option. How many data should this collection have? In this project, the necessary power of the electrolyzer was calculated based on kilowatts or kilojoules per second. The basis of production should also be 5 kg of hydrogen per day. As a result, 86,400 s and its equivalent, the numbers for the wind speed should be created. But the intermittent nature of the wind caused the time frame to change from daily to yearly. This decision has several advantages. First, the members of the dataset increase greatly; Based on the probability theory of the Law of large numbers, this causes the average of created numbers to be closer to the number chosen as the average or the shape parameter. That is, the mean of the dataset becomes closer to the expected value as the population increases^32^. Another thing is that, by considering the annual interval, the simulation of the dataset can be more adapted to nature. The gamma distribution function cannot accept zero values, but on the other hand, there are days in nature when the wind doesn't blow, so the wind speed must be considered zero. To make this change, it is enough to assume that the annual dataset is smaller. For this project, 20% of days of the year without wind were assumed. As a result, the dataset with these assumptions should have 25,246,080 members. If we wanted to include this value on a daily basis, the dataset would be small and its average value would be far from the expected value.

Ten datasets, with natural numbers from 1 to 10 as mean, were created by the method described above and with the help of the gamma distribution function. The reason for choosing these ten numbers was the only convenience in calculations, otherwise, there is no preference between the number 1 and 1.265, for the average wind speed for a point on the earth. As shown in Fig. 2d, the map of the average wind speed at 100 m height, from 2.5 to 10 m/s, and its range is specified globally. As can be seen from this map, the average wind speed can take any value. To have an overview of these ten datasets, in Fig. 1, their histogram and density plot are displayed simultaneously in one plot. Each of these datasets was fed into the algorithm to determine which average wind speed could satisfy the project's needs.

#### Calculation of turbine output power

To calculate the power output from the turbine, the equation of the amount of kinetic energy of the wind should be used. This equation is as follows^33^:

2 $${\mathrm{E}}_{\mathrm{ke}}=0.5 {\uprho }_{\mathrm{air}}\mathrm{ A }{\mathrm{v}}^{3}\mathrm{ t}$$

In Eq. (2), the term on the left is the kinetic energy of the wind, the first term on the right is the air density in the desired conditions, the second term is the surface against the wind flow, the third term is the speed of the wind flow, and finally, the last term is time. According to the definition of power, the amount of energy consumption or production per unit of time is equivalent to the power consumption or production, as a result, the amount of wind power is calculated according to the conditions of the wind turbine as follows^34^:

3 $${\mathrm{P}}_{\mathrm{T}}=0.5 {\mathrm{C}}_{\mathrm{P}} {\uprho }_{\mathrm{air}} {\mathrm{A}}_{\mathrm{T}} {\mathrm{v}}^{3}$$

In Eq. (3), C_P_ is the overall power coefficient of the turbine and, A_T_ is the area swept by the turbine rotor.

Equation (3) shows the necessary variables to calculate the amount of power output from the wind sector of the mini-Factory. The last term of the equation, i.e., wind speed, was fully discussed in the previous step. But how are the rest of the variables determined? The overall power coefficient is generally divided into two elements, the turbine rotor section, and the electrical section. In the case of the electrical part, which is mainly composed of the generator and the converter, a general coefficient of 0.9 was determined, which is usually adopted for these systems. But in the case of the turbine rotor, calculating the efficiency factor is a bit complicated. According to the equations mentioned in different sources^34–36^, rotor efficiency depends on factors such as tip speed ratio (TSR) and pitch angle. TSR itself depends on the instantaneous wind speed. On the other hand, regarding the pitch angle, for VAWTs, which are not normally equipped with a pitch angle adjustment system, this value changes instantaneously with the rotation of the turbine. With these interpretations, the complexities of the matter made the usual nominal efficiency for VAWTs be considered. This value is around 0.4, as mentioned in the Wind power generation system section. Finally, by multiplying these two values together, the overall power coefficient of the wind sector was determined as 0.36.

The next term, the air density, is obtained from the following equation, assuming that the air is an ideal gas and without taking into account the air humidity:

4 $${\uprho }_{\mathrm{air}}= \frac{\mathrm{P }{\mathrm{M}}_{\mathrm{air}}}{\mathrm{R T}}$$

In Eq. (4), P and T are respectively the same pressure and temperature of the environment, which were assumed to be similar to the environmental conditions of the electrolyzer. M_air_ is the molecular mass of air that was extracted from the sources. R is also the universal gas constant, which was determined to be 8.3144 [J/mol K].

The area swept by the turbine rotor is the same variable that should be determined by trial and error in this step of the algorithm. To begin, a value must be assumed for it. According to the classification of turbines based on dimensions and power, usually medium-scale turbines have a size of about 100 m^2^ or a power of about 100 kW^3^. Accordingly, to start the calculation, the surface of 100 m^2^ was considered as the initial assumption.

But what criteria are there to control the dimensions of the surface? In general, there are two aspects to limit the surface. As indicated in Fig. 5, these two aspects are the occupied space and the necessary costs. The occupied space is important because it follows the main purpose of this project. In this project, it is based on the fact that a wind turbine will eliminate the energy needs of a household. As a result, the space for the installation of a wind turbine is a space that is at the disposal of a household. For this reason, it should be as small as possible, so as not to disturb the routine life of the household. Necessary costs, assuming a direct relationship with dimensions, had control over the amount of surface. The bigger the surface, the cost of building the turbine, installation of it, and on the other hand, creating a proper foundation for those dimensions will increase. Since these two criteria do not have a specific numerical value to control the surface, they were implicitly included in the algorithm.

Another point that should be checked in this step is the turbine power curve. According to the turbine power curve, three effective speeds are defined for each wind turbine. These three wind speeds are cut-in speed, cut-out speed, and rated speed^37^. Cut-in speed, as mentioned in the Simulation of wind speed section, refers to the minimum speed that the turbine needs to move the rotor. That means, at speeds lower than that value, power is not produced, because the rotor doesn't move. Cut-out speed refers to the maximum speed at which, to prevent damage to the turbine, the rotor is separated from the generator. That means, at speeds higher than that value, even with the rotation of the rotor, power is not produced by the generator. The rated or the nominal speed of the turbine, is the speed that the turbine is designed for it. This means that the efficiency of the turbine is maximum in this state. As a result, more power is not produced by the turbine over this wind speed.

How can the changes caused by these speeds be applied to the output power of the turbine? First, the values of these speeds should be determined according to the type of turbine selected for this project. By examining different sources [^21,37^], the cut-in, cut-out, and rated speeds were selected as 4, 25, and 15 m/s, respectively. Then, using the <and> operators in the Python programming language, the speed dataset was filtered. That means, numbers smaller than 4 were equal to zero, numbers greater than 25 were equal to zero, and numbers greater than 15 were equal to 15. For all ten datasets created for average speeds of 1 to 10 m/s, these changes in speed were applied before calculating the turbine power, then the turbine power was calculated.

#### Calculation of electrolyzer power density

After calculating the final output power from the wind turbine, the amount of input power to the electrolyzer should be calculated. In the case of electrolyzers, power per unit area or power density is the main design element. In this way, the power of the electrolyzer is calculated based on the amount of power required on the active surface, which is obtained by multiplying the current density by the potential. As a result, before applying the input power, a value must be determined for the active surface of the electrolyzer.

Usually, in electrolyzer modeling, the active surface is assumed to be 1 m^2^, so that it doesn’t affect the calculations^38^. But, in this project, since the active surface is also effective in the amount of power applied to the electrolyzer, it must be optimized. So, in this way, 1 m^2^ was placed in the algorithm for the initial guess value of the active surface. But what is the limitation of this component? As can be seen from Fig. 5, the cost limit was included as a criterion to limit the surface increase. Since PEM electrolyzers are expensive due to the materials used in them^39^, the surface should be as small as possible to reduce the purchase cost of the electrolyzer. Since there is no numerical limit for this criterion, this criterion was implicitly included in the algorithm.

#### Calculate the resulting current density

The dependence of power density on current density is a bit complicated. The power density is obtained by multiplying the current density by the actual cell potential. On the other hand, the actual cell potential also depends on the current density. Several equations are involved in this path, which makes the task of finding the current density from the power density long and complicated. All these equations are given in full in the next section, Thermodynamic aspects of the mini-Factory.

The solution adopted in this project to reduce the complexity was that, at the beginning of the work, a series of numbers, from 0 to 20,000 with a step of 10, was created as current density values. Then, this series was fed into the corresponding equations, so that finally, for each current density value, a corresponding power density value was created. Then, these two sets of numbers were depicted in a graph as explanatory and response variables, so that the appropriate model can be guessed to create an equation between these two variables. According to the graph as well as sources^38^, the relationship between current density and power density is a linear relationship. So, by applying linear regression, the following equation was created between two variables:

5 $${\mathrm{P}}_{\mathrm{Elc}}=0.0021 {\mathrm{J}}_{\mathrm{Elc}} -0.5438$$

In Eq. (5), the power density is in kW and the current density is in A/m^2^. By inverting this equation, the corresponding current density can be found from the power densities calculated in the previous step. In this step, as explained in detail in the PEM electrolyzers, a good partner for the dynamic systems section, an explicit value of 20,000 A/m^2^ was created for the current density ceiling^30^. This provides an implicit criterion for electrolyzer efficiency as well.

#### Calculation of mass of produced hydrogen

From the relationship between current density and mass transfer rate, it is possible to calculate the amount of hydrogen produced based on the current density applied to the electrolyzer. Its equation is as follows^40^:

6 $${\mathrm{N}}_{\mathrm{j}} = \frac{\mathrm{J}}{{\mathrm{m}}_{\mathrm{j}}\mathrm{ F}}$$

In Eq. (6), N_j_, is the rate of production or consumption of species j in terms of mol/s m^2^, J is the current density, F is Faraday's constant, and m_j_ is a coefficient obtained based on the stoichiometric coefficients of the water decomposition reaction. It is equal to 2 for hydrogen and water and 4 for oxygen. As can be seen from the unit given for N_j_, the value of moles per unit area is obtained from this equation. Consequently, to find the mass of hydrogen produced, the value of N_j_, must be multiplied at the surface determined for the electrolyzer in the third step and the molecular mass of hydrogen. Finally, to find the total amount of produced mass during a year, all the values obtained from the wind speed dataset should be added together to get the final value.

The final total amount of produced hydrogen per year should be equal to 1825 kg, which is equivalent to 365 days multiplied by the daily production of 5 kg of hydrogen.

#### Calculation of additional energy required for heating water

In the Thermodynamic aspects of the mini-Factory section, it is explained in detail that the heat required to warm up the water entering the electrolyzer must be supplied with the help of a wind turbine, through an electric water heater. So, more power must be produced in the turbine to supply this energy. Since, without calculating the amount of produced hydrogen, the amount of water needed for hydrogen and then the heat needed to warm up it could not be calculated, as a result, in this project another approach was adopted to calculate this energy.

The approach of this project is that, after calculating the required amount of hydrogen, i.e., the same amount of 1825 kg, the mass of required water for this amount of hydrogen was determined. Then, based on the thermodynamic conditions of the input and output of the electric water heater, the amount of needed heat to warm up this mass of water was determined. Because, in electric water heaters, the input electric power is directly transferred to the water^41^; It is possible to find the amount of electrical power with the help of the amount of required heat, after dividing by the efficiency of the water heater and multiplying by 1.1 for brine water. In the next step, the calculated power value for the electric water heater was added with the required power for the electrolyzer, then, this total value, the new output power, was assumed for the wind turbine. As a result, with the help of this newly defined power and the wind speeds specified in the first step, the final modified required surface for the turbine was calculated.

#### Step-by-step implementation of the mentioned stages

With initial guess values for the turbine surface and the electrolyzer surface, from the 1 m/s average wind speed dataset, the algorithm began to calculate. The wind speed of 1 m/s did not satisfy the purpose of this project due to the removal of large amounts of data with the cut-in speed limit. As a result, the next speeds were tried in order. Finally, after reaching the average speed of 7 m/s, this dataset seemed suitable. In Fig. 4, this dataset with the average wind speed of 7 m/s is shown in four periods. So, the final optimization process for the two desired surfaces began.

The final calculations made obtained a suitable surface of 127 m^2^ for the wind turbine. For Darius H-shaped VAWT, according to sources, the most appropriate ratio of blade height to rotor radius is equal to 2.8^42^; As a result, the surface swept by the turbine becomes a vertical rectangle of approximately 10 × 13 m. For a HAWT, the blade length for this surface is equal to 6.3 m.

After the calculations for the surface of the electrolyzer, the desired surface was equal to 2.25 m^2^. Assuming cells with active surface dimensions of 25 × 25 cm (area of 0.0625 m^2^), 36 cells with these dimensions are needed to meet the need for hydrogen production.

The overall dimensions of the system seem favorable for the conditions of a mini-Factory, but the average wind speed is a bit high. This happens for various reasons; First, in the calculations, the wind speed was assumed to be zero for 20% of the year. Second, turbines cannot convert all wind energy into electrical energy. Third, the relationship between turbine power and wind speed is a 3rd-degree relation, as mentioned in Eq. (3). This causes that with an increase of one unit in the speed, the power increases by the power of three, as a result, the difference between a certain speed and its next and previous speed shows a drastic difference in the production power.

Now how can the mini-Factory be developed for other places with less average wind speed? One of the solutions, as mentioned in the Simulation of wind speed section, is to increase the height of the turbine. The wind gradient equation is defined as follows^43^:

7 $${\mathrm{V}}_{\mathrm{H}} = {\mathrm{V}}_{10} { \left(\frac{\mathrm{H}}{{\mathrm{H}}_{10}}\right)}^{\mathrm{\alpha }}$$

In Eq. (7), V_H_ and V_10_ are respectively the wind speed at the height of H and 10 m from the ground in m/s, H and H_10_ are respectively the height of H and 10 m from the ground and alpha is Hellmann exponent is different based on the terrain. This value has a value of 0.34 for conditions similar to my assumption, i.e., neutral air above human-inhabited areas^44^. As a result, assuming a wind speed of 7 m/s at height H, and setting speeds less than 7 m/s for a height of 10 m, the height change required to reach a wind speed of 7 m/s was calculated, which is shown in Table 1.

For example, for an average wind speed of 6 m/s at a height of 10 m, you must go to a height of 16 m to experience a wind speed of 7 m/s. This height change is also possible with VAWT for a speed of 5 m/s, but for speeds of 4 and 3 m/s, you should choose the HAWT. For an average wind speed of 2 m/s, airborne wind systems seem appropriate, but for a wind speed of 1 m/s, there is currently no technology to harvest wind energy at an altitude of 3000 m. Of course, it should be accepted that Eq. (7) is an approximate equation and the influencing factors must be determined depending on the conditions of each point.

#### Evaluating the costs of manufacturing the Hydrogen mini-Factory

Now that the dimensions of the system components are obtained, it is possible to calculate the cost of building the Hydrogen mini-Factory. But what are the challenges ahead?

In the calculation of the electrolyzer, it was assumed that the hydrogen produced goes out of the electrolyzer and is stored under atmospheric conditions. By searching online sales websites, you will find that the market of PEM electrolyzers focuses only on electrolyzers with high working pressure. This makes the design of this type of electrolyzer more expensive than atmospheric working pressure; Because special materials and design are used to withstand high pressure. On the other hand, the price range of electrolyzers is wide based on their quality, and it is not possible to get an exact price for this product. However, roughly, based on existing products, you should pay $40,000 per 0.5 m^2^ of electrolyzer active surface. According to the design dimensions of the mini-Factory, the calculated active area is 2.25 m^2^, which with a simple ratio, its price is $180,000.

This price is costly, but as mentioned, it's for high-pressure conditions, no doubt, for atmospheric conditions, this cost will be lowed; But there is a need to redesign the existing electrolyzers for atmospheric pressure.

Now let's go to the wind section. The wind sector has more complex challenges. Firstly, there are no specific Darrieus straight-bladed wind turbines on online sales websites. HAWTs are available in a wide price range, from household sizes to wind farm sizes. But in the case of VAWT, there are Helical H-rotors, helix maglev, and combined designs of Savonius and Darrieus. As a result, the closest design to this project is the Helical H-rotor. Another point is that wind turbines are sold as a complete package, including the rotor and generator. On the other hand, the generators used in them are also, normally, axial flux or coreless. As a result, it is not compatible with the design of this project, which uses PMSGs.

With all these challenges, based on current prices, we have to pay $100 per 1 m^2^ of surface covered by the turbine. The total area of the wind turbine for the mini-Factory was calculated to be 127 m^2^, which makes the total price of the wind system equal to $12,700.

The costs of the heat transfer part, including a 1.3 kW heat exchanger and a 50 W electric water heater, are approximately $50 in total.

A 300-L LLDPE water tank costs $30 to store water for a week of mini-factory activity.

In the last step, I investigate hydrogen storage. The aim of this project is to evaluate the possibility of wind energy being suitable for meeting the household's energy needs. But the amount and time of wind energy supply often will not coincide with the demand for energy; For this purpose, the need for a storage system is felt. In this project, hydrogen was chosen as an energy carrier and reservoir; Because compared to the usual methods of electricity storage, such as batteries, which can only store electricity for a short time, it is a suitable option. Depending on the chosen method of hydrogen storage, it can be stored even for months without any loss. But, the more important point, before considering the storage method, is its consumption method. Hydrogen is a versatile carrier; It can be used as gas in the oven to cook food; It can be used as electricity produced by a fuel cell; Or it can be stored compressed in the car tank as fuel. It depends on the household itself which method to choose.

Hydrogen storage methods are developing so rapidly that it is impossible to choose the best method for storage at this time. The premise of this project was storage in atmospheric conditions, which, to store the produced hydrogen by the mini-Factory, during a week, that is, equivalent to 35 kg, a space of about 392 m^3^ is required. It is completely unreasonable to want to store hydrogen in such conditions. One of the most interesting solutions is storing hydrogen in aqueous solutions based on edible salts such as baking soda. This method, which is based on the bicarbonate-formate cycle, is an attractive way to store hydrogen in a cheap way using salts found in abundance in nature. According to the research of Gutiérrez et al.^45^, with the help of this method, it is possible to store 27 g of hydrogen per 1 L of solution; This means that 1260 L of solution or 1.26 m^3^ of space are needed to store 1 week's production. Compared to the gas storage method, the difference is really impressive. Due to the fact that common salts are used for storage in this way, it can be said that the cost of storage is not high; However, to perform the bicarbonate-formate cycle process, Pd/C catalyst is needed, which increases the cost of this storage system. Consequently, with an estimate for hydrogen storage, I assume $5000 for 1 week.

Finally, with additional costs, such as the foundation for the VAWT, the approximate total cost of the Hydrogen mini-Factory is $200,000.

The cost of producing 1 kg of hydrogen, with the help of renewable energy, is around $5 (https://www.energy.gov/eere/fuelcells/hydrogen-shot). Assuming that this price remains constant and taking into account the annual production of 1825 kg of hydrogen by the mini-Factory, the investment payback period for this project will be 22 years. It is not an attractive option from the point of view of investment, but it can be attractive for households from the point of view that the household doesn't need to pay for electricity, gas, and car fuel.

### Thermodynamic aspects of the mini-Factory

All thermodynamic systems are based on the quadruple equations of mass, energy, entropy, and exergy. Electrolyzers and wind turbines are no exception to this rule. For this purpose, at the beginning of the path, these quadruple equations should be mentioned. Since, in this project, all systems have mass transfer in addition to energy transfer, so by definition, they are open systems. The general form of the quadruple equations of mass, energy, entropy, and exergy for an open system is as follows^46^:

8 $${\mathrm{MBE}{:}\;\dot{\mathrm{m}}}_{\mathrm{in}}= {\dot{\mathrm{m}}}_{\mathrm{out}}$$

9 $${\mathrm{EBE}{:}\; \dot{\mathrm{m}}}_{\mathrm{in}}{\mathrm{ h}}_{\mathrm{in}}+ {\dot{\mathrm{Q}}}_{\mathrm{in}}= {\dot{\mathrm{m}}}_{\mathrm{out}} {\mathrm{h}}_{\mathrm{out}}+ {\dot{\mathrm{W}}}_{\mathrm{out}}$$

10 $$\mathrm{EnBE}{:}\; {\dot{\mathrm{m}}}_{\mathrm{in}} {\mathrm{s}}_{\mathrm{in}}+ {\dot{\mathrm{Q}}}_{\mathrm{in}}/{\mathrm{T}}_{\mathrm{S}}+{\dot{\mathrm{S}}}_{\mathrm{gen}}= {\dot{\mathrm{m}}}_{\mathrm{out}} {\mathrm{s}}_{\mathrm{out}}$$

11 $$\mathrm{ExBE}{:}\; {\dot{\mathrm{m}}}_{\mathrm{in}} {\mathrm{ex}}_{\mathrm{in}}+ {\dot{\mathrm{Q}}}_{\mathrm{in}}(1- {\mathrm{T}}_{0}/{\mathrm{T}}_{\mathrm{S}})= {\dot{\mathrm{m}}}_{\mathrm{out}} {\mathrm{ex}}_{\mathrm{out}}+ {\dot{\mathrm{W}}}_{\mathrm{out}} + {\dot{\mathrm{Ex}}}_{\mathrm{d}}$$

In the quadruple equations above, the first equation is the mass balance equation (MBE), the second equation is the energy balance equation (EBE), the third equation is the entropy balance equation (EnBE) and the fourth equation is the exergy balance equation (ExBE).

In the quadruple Eqs. (8), (9), (10), and (11), the variables with subscripts in and out are related to the input and output flows of the system. The variables with the dot symbol on them indicate the rate of the variable, which means that the symbols m, Q, W, S_gen_, and Ex_d_, are respectively, mass flow rate, heat transfer rate, work transfer rate, entropy production rate, and exergy destruction rate. Variables with lowercase letters, such as h, s, and ex, are symbols of specific enthalpy, specific entropy, and specific exergy of the flow, respectively. The symbols T_0_ and T_S_ are also related to the reference temperature and the temperature of the heat source.

With the help of these equations, it is possible to calculate the amount of required heat or the produced work, or the exergy destruction of each part of the system. Specific enthalpy and entropy values for most pure substances are available in sources, but specific exergy must be calculated using the following equation. This equation is related to the calculation of specific exergy flows for an open system^46^:

12 $$\mathrm{ex}=\left(\mathrm{h}- {\mathrm{h}}_{0}\right)- {\mathrm{T}}_{0} (\mathrm{s}- {\mathrm{s}}_{0})$$

In Eq. (12), the symbols with subscript 0 correspond to the reference conditions and the symbols without subscript correspond to the conditions of the flow.

Now let's go to the electrolyzer. To calculate the necessary power density, we use a relationship similar to electrical work, i.e., the product of the current multiplied by the potential. The power density equation for electrolyzers is as follows^47^:

13 $$\mathrm{P }=\mathrm{ E}*\mathrm{J}$$

In Eq. (13), the first term is the power density in W/m^2^, the second term is the cell potential of the electrolyzer in V, and the third term is the current density in A/m^2^.

The electrolysis cell potential can be calculated through the following equations, with the help of current density and operating parameters. As mentioned in the previous section, for this project, the amount of current density is determined by the amount of power density and power output from the turbine and as a result the wind speed. This means that for each wind speed created with the help of gamma distribution function, a turbine power, then an electrolyzer power density, and finally a current density was calculated respectively. Eventually, with the help of this current density, the final mass of produced hydrogen was calculated.

The actual cell potential, or cell potential of the electrolyzer, is calculated from the sum of the standard potential and overpotentials. The standard potential, which is directly dependent on the water electrolysis reaction and operating conditions, but the overpotentials are based on the conditions of the electrodes and electrolyte. In general, the equation for calculating the actual cell potential is as follows^38^:

14 $$\mathrm{E}={\mathrm{E}}_{\mathrm{r}}+{\upeta }_{\mathrm{act}}+ {\upeta }_{\mathrm{ohm}}$$

In Eq. (14), the first term is the actual cell potential, the second term is the reversible cell potential, the third term is the activation overpotential, and the last term is the ohmic overpotential. In the calculation of the actual cell potential electrolyzers, there is another term called concentration overpotential, which in the case of PEM electrolyzers, due to its low value, is not included in the calculations at current densities of less than 20,000 A/m^2^.

To calculate the reversible cell potential, the following equation should be used^48^:

15 $${\mathrm{E}}_{\mathrm{r}}= {\mathrm{E}}_{0}+ \frac{\mathrm{R T}}{\mathrm{n F}}\mathrm{ ln} \left (\frac{{\mathrm{P}}_{\mathrm{H}_{2}} {\mathrm{P}}_{\mathrm{O}_{2}}^{0.5}}{{\mathrm{P}}_{\mathrm{H}_{2}\mathrm{O}}}\right)$$

In Eq. (15), E_0_ is the reversible potential of the cell in standard conditions (T_0_ = 289.15 K and P_0_ = 101 kPa). The change in operating temperature and pressure directly affects the water electrolysis reaction. For this purpose, with the help of the first term on the right side of the equation, this change in the amount of potential should be included. On the other hand, for the effect of the concentration of the participants in the water electrolysis reaction, the second term on the right side of the equation is used to correct the actual cell potential value. In the second term, R is the global gas constant equal to 8.3145 J/mol K, F is Faraday constant equal to 96,485 C/mol, n, is the number of consumed electrons per mole of produced hydrogen in the electrolysis cell, which is equal to 2 here, and T, the operating temperature assumed in this project is equal to 353 K (80 °C). The variable P with the subscripts H_2_, O_2_, and H_2_O respectively is the partial pressure of the three substances involved in the water reaction, i.e., hydrogen, oxygen, and water. Of course, in this equation, instead of partial pressure, the molar fraction of the material is used, for this reason, it was assumed that for two gases, hydrogen, and oxygen, which are produced separately on both sides of the electrolyzer, it is equal to one, and for water, which is in the liquid phase, is also equal to one.

The value of the standard potential varies with temperature and pressure, depending on the operating conditions. In this project, the operating pressure was assumed to be equal to the standard condition, i.e., 100 kPa. But the operating temperature, as mentioned, was considered to be 80 °C; As a result, the standard potential should be calculated with new conditions. This temperature change can be calculated using the following equation^48^:

16 $${\mathrm{E}}_{0}=1.229-0.0008464 (\mathrm{T}-298.15)$$

In Eq. (16), the value of 1.229 is the minimum required potential under standard conditions based on the water electrolysis reaction. This value should decrease linearly with increasing temperature. As a result, this effect appears in the second term of the right side of the equation.

The next step is to calculate the activation potential. For this overpotential, the Butler-Volmer equation should be used. This equation, which is based on the overpotential of the cathode and anode as the explanatory variable and the current density as the response variable, after inverting it, becomes as follows^30^:

17 $${\upeta }_{\mathrm{act},\mathrm{ i}}= \frac{0.5\mathrm{ R T}}{{\mathrm{\alpha }}_{\mathrm{i}}\mathrm{ F}} {\mathrm{sinh}}^{-1} \left(\frac{\mathrm{J}}{{2\mathrm{ J}}_{0,\mathrm{i}}}\right)$$

R, T, and F parameters in Eq. (17), were explained in Eq. (15). The α_i_ parameter is charge transfer coefficients, which according to the sources were assumed equal to 0.5 for both the cathode and anode. J is also the current density and J_0,i_ is the exchange current density. The J_0,i_ variable depends on the activation energy and temperature, but since in this project, the conditions were modeled similarly to the conditions of other articles, the calculated parameters were used directly.

In the next step, we go to ohmic overpotential, which is more complicated to calculate. Ohmic overpotential is basically the resistance of the proton exchange membrane against the hydrogen ions that pass through it and move from the anode to the cathode.

In general, the equation for calculating this overpotential is as follows^38^:

18 $${\upeta }_{\mathrm{ohm}}=\mathrm{J}* {\mathrm{R}}_{\mathrm{PEM}}$$

To calculate the value of resistance R_PEM_, the following equation should be used^38^:

19 $${\mathrm{R}}_{\mathrm{PEM}}= {\int }_{0}^{\mathrm{L}}\frac{\mathrm{dx}}{\upsigma [\uplambda \left(\mathrm{x}\right)]}$$

In Eq. (19), the amount of membrane resistance is calculated based on the integration over the movement path of ions, that is, the same amount of membrane thickness, which is L here. In the denominator of the integral fraction, there is σ that local ionic conductivity, which itself is a function of λ, the water content of the membrane, at point x of the length L. To calculate the water content of the membrane, the following equation should be used^38^:

20 $$\uplambda \left(\mathrm{x}\right)= \frac{{\uplambda }_{\mathrm{a }}- {\uplambda }_{\mathrm{c}} }{\mathrm{L}}\mathrm{ x}+ {\uplambda }_{\mathrm{c}}$$

In Eq. (20), λ_a_ and λ_c_ are respectively the water content at the interface between membrane-anode and membrane-cathode, which were determined from the sources of their value. The value of x and L is also replaced in terms of meters in this equation.

After calculating the water content, the local ionic conductivity can be found by inserting it into the following empirical equation^38^:

21 $$\upsigma \left[\uplambda \left(\mathrm{x}\right)\right]=\left[0.5139\uplambda \left(\mathrm{x}\right)-0.326\right]\mathrm{* exp} \left[1268 \left(\frac{1}{303}-\frac{1}{\mathrm{T}}\right) \right]$$

In Eq. (21), T is the membrane temperature. As a result, in general, the resistance of the membrane depends on three factors: temperature, thickness, and humidity, or the water content of the membrane.

Now that all the terms of Eq. (14) have been calculated, the remaining components of the electrolyzer can be calculated.

The entropy generation in the electrolyzer is directly related to the irreversible potential of the cell. This means that overpotentials, which are irreversible cell potentials, determine the amount of entropy generation in the electrolyzer. For this purpose, the following equation is used to calculate entropy generation^38^:

22 $${\mathrm{S}}_{\mathrm{gen}}=\frac{\mathrm{nF}}{\mathrm{T}} ({\upeta }_{\mathrm{act}}+ {\upeta }_{\mathrm{ohm}})$$

Equation (22) calculates the amount of entropy generation by the electrolyzer in terms of moles of hydrogen produced; As a result, to calculate the entropy generation rate, it is enough to multiply this value by the molar flow rate of the produced hydrogen.

The entropy generation is associated with an increase in heat in the system, so by finding the difference between the heat of the water electrolysis reaction and the heat caused by the entropy generation, you can find out whether the electrolyzer needs additional heat or not. This trade-off is expressed in the form of the following equation^38^:

23 $$\mathrm{Q}=\frac{\mathrm{J}}{\mathrm{n F}} (\mathrm{T}* (\mathrm{\Delta s }- {\mathrm{S}}_{\mathrm{gen}}))$$

To calculate the amount of heat caused by the reaction, the Δs term has been added to Eq. (23), so that by determining the sign of this difference, the need for heat can be determined. To calculate the entropy caused by the reaction, the amount of entropy of the substances participating in the water electrolysis reaction at the operating temperature should be determined in the following way^48^:

24 $$\mathrm{\Delta s}={[\mathrm{s}}_{\mathrm{H}_{2}}+ {0.5\mathrm{ s}}_{\mathrm{O}_{2}}]- {\mathrm{s}}_{\mathrm{H}_{2}\mathrm{O}}$$

As it was adopted from sources, PEM electrolyzers do not need additional heat and provide all the energy necessary for the electrolysis of water with the help of electrical energy. In this project as well, for all the samples created for current density, the amount of heat was always negative.

Since the amount of heat became negative, as a result, heat is removed from the system and the exergy due to this heat is calculated as follows:

25 $$\mathrm{Ex}=\mathrm{Q } \left(1- \frac{{\mathrm{T}}_{0}}{\mathrm{T}} \right)$$

In Eq. (25), T and T_0_ are the electrolyzer temperature and the reference temperature, respectively.

The following equation can be used to calculate the entropy caused by the heat of the electrolyzer:

26 $$\mathrm{S}=\mathrm{ Q }/\mathrm{ T}$$

Finally, with the help of the entropy generation rate calculated from Eq. (22), the amount of exergy destruction rate in the electrolyzer can be calculated with the help of the following equation^49^:

27 $${\mathrm{Ex}}_{\mathrm{d }}= {\mathrm{T}}_{0 }* {\mathrm{S}}_{\mathrm{gen}}$$

After calculating the thermodynamic elements of the electrolyzer, it is necessary to calculate its efficiency. There are two scenarios for the electrolyzer process. The first scenario that occurs at the beginning of the operation is when the electric water heater must raise the temperature of the water to vaporization, and then this saturated vapor is fed into the electrolyzer after cooling to the operating temperature of the electrolyzer and liquefaction. In this scenario, all the required power for water evaporation is provided by the electric water heater, in other words, the wind system. For this reason, the efficiency of the electrolyzer section is somewhat reduced, because the heat required to evaporate water is high.

For the second scenario, the two streams of warm gas exiting from the electrolyzer, as well as hot steam flow exiting from the electric water heater, can act as a heat source and reduce the amount of required heat by the electric water heater. In this situation, it is enough for the electric water heater to raise the water temperature to about 322 K. This amount of heat is much less compared to the required heat to bring water to the condition of saturated steam.

The exergy efficiency of the electrolyzer, like the energy efficiency, depends on the two elements of the required work for the electrolyzer itself and the required work to heat the water. Since, the required work for the electrolyzer and the work of the electric water heater are both electrical and are calculated from the relationships between the variables of resistance (R), potential (V), and current (I); As a result, to determine their exergy, the same amount of work in kilowatts should be used in the exergy equation.

In general, the energy efficiency equation of the electrolyzer is as follows:

28 $${\upeta }_{\mathrm{Elecr}}= \frac{{\dot{\mathrm{m}}}_{\mathrm{H}_{2}} {\mathrm{LHV}}_{\mathrm{H}_{2}}}{{\dot{\mathrm{W}}}_{\mathrm{Elecr }}+ {\dot{\mathrm{W}}}_{\mathrm{EWH}}}$$

Also, the exergy efficiency of the electrolyzer is obtained from the following equation:

29 $${\uppsi }_{\mathrm{Elecr}}= \frac{{\dot{\mathrm{m}}}_{\mathrm{H}_{2}} {\mathrm{ex}}_{\mathrm{H}_{2}}}{{\dot{\mathrm{W}}}_{\mathrm{Elecr }}+ {\dot{\mathrm{W}}}_{\mathrm{EWH}}}$$

In Eqs. (28) and (29), in the denominator of the fractions, the first term is the required work rate for the electrolyzer and the second term is the work rate of the electric water heater. Also, LHV_H2_ is the lower heating value of hydrogen, and ex_H2_ is the amount of total specific exergy, which is obtained from the following equation^49^:

30 $${\mathrm{ex}}_{\mathrm{H}_{2}}= {\mathrm{ex}}_{\mathrm{H}_{2}}^{\mathrm{ph}}+ {\mathrm{ex}}_{\mathrm{H}_{2}}^{\mathrm{ch}}$$

In Eq. (30), on the right side of the equation, the first term is the specific physical exergy of hydrogen, which is calculated from Eq. (12), and the second term is the specific chemical exergy of hydrogen, which was extracted from sources.

In the last step, the overall energy and exergy efficiency of the system should be determined. For this purpose, one should look at the definition of overall efficiency. The overall efficiency of the system is equal to the amount of output or production energy, divided by the amount of input or consumption energy. Since the goal of the mini-Factory is to produce hydrogen and its only output is hydrogen, then the amount of produced energy is equal to the numerator of the fraction of Eqs. (28) and (29). But what is the input energy to the system?

For this project, only one wind turbine is responsible for receiving energy from the airflow. The input energy to the area swept by the wind turbine is the total energy input to the system. So, to calculate the input total energy to the system, the amount of kinetic energy of the wind and then its power should be found with the help of Eq. (2).

In this project, it was assumed that the only energy that can be converted by the wind turbine is kinetic energy, and since heat is not transferred in this process, the amount of exergy was assumed to be the same as the amount of energy, similar to when work is transferred. Finally, with these conditions, the overall energy efficiency of the system is equal to:

31 $${\upeta }_{\mathrm{OV}}= \frac{{\dot{\mathrm{m}}}_{\mathrm{H}_{2}} {\mathrm{LHV}}_{\mathrm{H}_{2}}}{{\mathrm{P}}_{\mathrm{Wind}}}$$

Also, the overall exergy efficiency of the system is equal to:

32 $${\uppsi }_{\mathrm{OV}}= \frac{{\dot{\mathrm{m}}}_{\mathrm{H}_{2}} {\mathrm{ex}}_{\mathrm{H}_{2}}}{{\mathrm{P}}_{\mathrm{Wind}}}$$

Finally, according to the calculations, the overall energy and exergy efficiency of the system is equal to 28% and 27%, respectively. The reason why energy efficiency is lower than exergy efficiency is that hydrogen has a lower amount of specific chemical exergy than its LHV in storage conditions (the amount of specific physical exergy of hydrogen is zero due to its storage in reference or ambient conditions).

The overall low efficiency of the system originates from the fact that the wind turbine has many limitations for converting wind power. One of these limitations is the cut-in, cut-out, and rated speeds that cause the removal of wind speed values in various conditions.

### Statistical aspects of design

Calculations based on statistics and probability are always mixed with uncertainty. In this project as well, this issue is quite evident. These uncertainties can cause confusion in reporting the calculated numbers. For this purpose, a logical framework should be created to present the calculated numbers, so that an orderly structure is formed. Accordingly, in this section, step by step, the cases where the aspects of statistics and probability have been introduced to the project have been reviewed.

The first step and at the beginning of the work, the generation of random numbers, was done with the help of Python programming language. These are random numbers that as a substitute for wind speed; So, should be in a certain distribution, thus that they can be a good representative of the wind speed in nature. In this project, the gamma distribution function seemed to be a good choice, the reasons for which are detailed in the Simulation of wind speed section. Another point is that the data created is in standard form, and there is no change in the scale and location of the data on the x-axis. This change could be done with the help of loc and scale parameters in the gamma function, from the stats module of the scipy library (https://docs.scipy.org/doc/scipy/reference/generated/scipy.stats.gamma.html), but since these distributions eliminated the need of the project approximately, no change was needed. Because the data was created randomly, the random_state parameter was used to store this data and not change it. This parameter creates a default state for storing data that is generated for the first time. In this way, constant data was always used so that the calculations do not change.

The next step, when statistics came to the aid of this project, was to establish a relationship between the current density and the power density of the electrolyzer. Since the relationship between these two variables was similar to a linear relation, a linear regression model was used to create it. Its equation is the same as Eq. (5) which was given in Calculate the resulting current density section. The R^2^ and RMSE errors for the created model were 0.999 and 0.178, respectively. This level of accuracy shows the high adaptation of the model to the real data. But again, there are differences. The plotted graphs of the actual values and model-predicted values show that the graph of the actual values is a convex function. This graph is slightly higher than the line created with the help of the model at the beginning and end of the drawn range (0 to 20,000 A/m^2^ for current density) and is slightly lower in the middle of this range. As a result of this difference and according to the distribution of data, uncertainty and error are introduced into the calculations. So, this error should be fixed.

This is where the differences occur. The relationship between the variables, in most cases, is not linear, and it is not possible to check the calculations with the help of a representative of the total data, such as the average. That is, it cannot be said that the average of the generated dataset for the current density, multiplied by the average of the generated dataset for the actual cell potential, yields the average of the generated dataset for the power density. This is the reason that, at the beginning of the work, it was pointed out that a specific framework should be created for data reporting.

This general framework states that two types of approaches should be defined for presenting data. As explained in the Allocation of wind speed section, the total number of data required for this project is equivalent to the number of seconds in a year and is equal to 31,557,600. But, because at the beginning of the work, 20% of the days of the year were assumed to be without wind (zero wind speed), the number of created data was equal to 25,246,080. Here, these two approaches are defined as follows. Variables that should be reported directly from the average of the dataset created for it. This method can be easily implemented with the help of the mean method from the numpy library. Another approach is the variables that depend on the time period; That means, they should be averaged over the whole year. This method is achieved by using the sum method on the dataset and dividing the sum value by 31,557,600.

So, for variables that are not dependent on time, the first approach was used to report them. The actual cell potential is one of these variables because it is calculated based on the current density data, and it doesn't matter how much data there are. But variables such as the mass of produced hydrogen, which must be clearly defined in the time period, were used from the second approach to report them. All these variables were marked with asterisk in Tables 1, 2, and 3 in order to determine which approach was used to report each of these variables (Data Availability).

## Data Availability

All the calculations of this project were done with the help of Python programming language, which can be found at 10.5281/zenodo.8302211. All the thermodynamic data used in this project were obtained from the website of the National Institute of Standards and Technology (NIST). (https://webbook.nist.gov/chemistry/fluid/). All the data extracted from different sources along with their references are given in Table 1. All calculations performed for clarity and ease of access are given in Tables 1, 2, and 3.
