# Author Correction: Hydrogen mini-Factory for domestic purposes (wind version)

**DOI:** 10.1038/s41598-023-42523-1

**Published:** 2023-09-14

**Authors:** Dany Azig

**Affiliations:** Faculty of Environment, University of Tehran, Tehran, Iran

Correction to: *Scientific Reports* 10.1038/s41598-023-40205-6, published online 12 August 2023

The original version of this Article contained an error, where the Python code that supports the Methods section was added as Supplementary Information file. The code is now available in the Data Availability section.

As a consequence, in the Methods section,

“The written script is available as a file with .py extension as Supplementary Information 1.”

now reads:

“The written script is available in the Data Availability section.”

And,

“All these variables were marked with asterisk in Tables 1, 2, and 3 in order to determine which approach was used to report each of these variables (Supplementary Information).”

now reads,

“All these variables were marked with asterisk in Tables 1, 2, and 3 in order to determine which approach was used to report each of these variables (Data Availability).”

Furthermore, the Data Availability,

“All the calculations of this project were done with the help of Python programming language, which is given in a single integrated file (Supplementary Information 1). All the thermodynamic data used in this project were obtained from the website of the National Institute of Standards and Technology (NIST). (https://webbook.nist.gov/chemistry/fluid/). All the data extracted from different sources along with their references are given in Table 1. All calculations performed for clarity and ease of access are given in Tables 1, 2, and 3.”

now reads:

“All the calculations of this project were done with the help of Python programming language, which can be found at https://doi.org/10.5281/zenodo.8302211. All the thermodynamic data used in this project were obtained from the website of the National Institute of Standards and Technology (NIST). (https://webbook.nist.gov/chemistry/fluid/). All the data extracted from different sources along with their references are given in Table 1. All calculations performed for clarity and ease of access are given in Tables 1, 2, and 3.”

The original incorrect Supplementary Information files appear below.

Lastly, the original version of this Article was revised: In the original version of this article, the ORCID ID for the author Dany Azig was omitted. The ORCID ID for Dany Azig is 0000-0002-2166-539X.

The original Article has been corrected.

### Supplementary Information

Supplementary Information 1.

Supplementary Information 2.

