# Supplementary material for: Author Correction: Hydrogen mini-Factory for domestic purposes (wind version)
Source: Sci Rep. 2023 Sep 14;13:15214. doi: 10.1038/s41598-023-42523-1 (PMC10502135; doi:10.1038/s41598-023-42523-1)
Supplement: Supplementary file 1 — Supplementary Information 1. [file 41598_2023_42523_MOESM1_ESM.docx]

#### Hydrogen mini-Factory (Wind-Version) ####

# author: Dany Azig

# Contact: dany.azig.96i@gmail.com

### Goal >>> Hydrogen mass flow rate = 5 kg/day /// Daily production of 5 kg of hydrogen by mini-Factory

## Energy and Exergy Efficiencies (Calculated) >>>

# Overall mini-Factory Energy efficiency = 0.27762240732253884

# Overall mini-Factory Exergy efficiency = 0.27028426814791684

# Imported Library:

import numpy as np

import pandas as pd

import seaborn as sns

import matplotlib.pyplot as plt

from scipy import stats

from math import log, exp, sqrt

## Defined Functions:

## Electrolyzer

# Standard Potential

def standard_potential(T):

standard_potential = 1.229 - 0.0008464 * (T - 298.15)

return standard_potential

# Reversible potential

def reversible_potential(E0, R, T, F, P0_H2, P0_O2, P0_H2O):

part1 = (P0_H2 * (P0_O2)**0.5) / P0_H2O

part2 = 0.5 * R * T / F

part3 = log(part1)

reversible_potential = E0 + part2 * part3

return reversible_potential

# Ohmic Overpotential

def ohmic_overpot(lambda_a, lambda_c, L_Elct, T, J):

part1 = (lambda_a - lambda_c) / L_Elct

part2 = 0.5139 * part1

part3 = 0.5139 * lambda_c - 0.326

part4 = exp(1268 * (1/303 - 1/T))

part5 = 1/(part4*part2) * log(abs((part2 * L_Elct + part3) / part3), 10)

ohmic_overpot = part5 * J

return ohmic_overpot

# Exchange Current Density

def exchange_current(gamma, E_act, R, T):

part1 = - E_act / (R * T)

exchange_current = gamma * exp(part1)

return exchange_current

# Activation Overpotential

def activation_overpot(R, T, F, J, J_0, alfa):

part1 = 0.5 * R * T / (alfa * F)

part2 = J / (2 * J_0)

activation_overpot = part1 * np.arcsinh(part2)

return activation_overpot

# Exergy

def exergy(h, h0, s, s0, T0):

part1 = h - h0

part2 = s - s0

exergy = part1 - T0 * part2

return exergy

## Wind system

# turbine power

def turbine_power(C_P, rho_Air, A, V):

turbine_power = 0.5 * C_P * rho_Air * A * V**3 / 1000

return turbine_power

# Wind Gradient

def height(WS, V10, a_H):

height = 10 * (Wind_Speed / V10)**(1/a_H)

return height

# Final Surface

def final_surface(Pow, C_P, rho_Air, V):

final_surface = (2 * Pow * 1000) / (C_P * rho_Air * V**3)

return final_surface

## Wind System ##

#Definition of gamma distribution function to determine wind values randomly

#25246080 >> 80% windy time of year

Wind_Speed = 7 #[m/s] @ 10 [m] height

WindSpeed = stats.gamma.rvs(a = Wind_Speed, loc=0, scale=1, size=25246080, random_state=1)

WindSpeedDF = pd.DataFrame(WindSpeed)

#print(WindSpeedDF.describe())

#Create graphs for the data generated for the desired wind speed

plt.rcParams['figure.figsize'] = [20,7]

plt.rcParams['figure.dpi'] = 300

#All of data (Annual)

WindSpeedDF.plot(legend=False, color='#EE82EE', marker='.', linestyle='', markersize=5)

plt.ylabel('Wind Speed [m/s]')

plt.xlabel('Time [Second]')

plt.title('Wind Speed in the entire defined time period')

plt.show()

#First Day

WindSpeedDay = WindSpeedDF[0:86400]

WindSpeedDay.plot(legend=False, color='#FF00FF', linewidth=0.5)

plt.ylabel('Wind Speed [m/s]')

plt.xlabel('Time [Second]')

plt.title('Wind Speed on the first day')

plt.show()

#First Hour

WindSpeedHour = WindSpeedDF[0:3600]

WindSpeedHour.plot(legend=False, color='#9400D3', linewidth=0.5)

plt.ylabel('Wind Speed [m/s]')

plt.xlabel('Time [Second]')

plt.title('Wind Speed in the first hour')

plt.show()

#First Minute

WindSpeedHour = WindSpeedDF[0:60]

WindSpeedHour.plot(legend=False, color='#4B0082', linewidth=4)

plt.ylabel('Wind Speed [m/s]')

plt.xlabel('Time [Second]')

plt.title('Wind Speed in the first minute')

plt.show()

#Density & Histogram plot of desired wind speed

plt.rcParams['figure.figsize'] = [12,7]

ax = sns.distplot(WindSpeed,

kde=True,

bins=100,

hist_kws={'linewidth': 2,'alpha':1, 'color':'skyblue', 'edgecolor':'gray'},

kde_kws={'linewidth': 5,'alpha':1, 'color':'darkblue'})

ax.set(xlabel='Distribution of Wind Speed values [m/s]', ylabel='Frequency')

plt.show()

#Alternative function for 'distplot' because this is a deprecated function and will be removed in a future version.

sns.histplot(WindSpeed,

kde=True,

bins=100,

color= 'skyblue',

edgecolor='gray',

alpha=1,

linewidth=2,

line_kws={'linewidth': 5, 'alpha':1, 'color':'darkblue'})

plt.xlabel('Distribution of Wind Speed values [m/s]')

plt.ylabel('Count')

plt.show()

# Calculation of total power for all generated wind speeds

# Ambient Conditions

T_env = 300 #[K]

P_env = 100 #[kPa]

#Constants

R_GC = 8.3144 #Gas Constant [J/mol-K]

# Wind turbine specifications

C_P1 = 1 # The total power available in the wind

M_Air = 0.0289652 #[kg/mol]

rho_Air1 = (P_env * 1000 * M_Air) / (R_GC * T_env) #[kg/m3]

#print(rho_Air1)

A_Final = 126.64794770180964 #Turbine swept area [m2] (Calculated)

V_air = WindSpeed

TurPowI = turbine_power(C_P1, rho_Air1, A_Final, V_air)

TurPowIDF = pd.DataFrame(TurPowI)

#print('Sum of the annual total power output ', sum(TurPowI)) # >> 935465165.7482802 [kW-year]

#Applying wind turbine operating limits to wind speed data

#cut-in speed = 4 [m/s]

#cut-out speed = 25 [m/s]

WindSpeed[(WindSpeed < 4) | (WindSpeed > 25)] = 0

#rated speed = 15 [m/s]

WindSpeed[WindSpeed > 15] = 15

# Calculation of turbine production power for all generated wind speeds

# Wind turbine specifications

C_Tur = 0.4 # Turbine efficiency for a typical VAWT

C_Gen = 0.9 # Efficiency of electric unit including generator and converter

C_P1 = C_Tur * C_Gen

A1 = 131 #Turbine swept area [m2] (Initial guesses)

V_air = WindSpeed

TurPow = turbine_power(C_P1, rho_Air1, A1, V_air)

TurPowDF = pd.DataFrame(TurPow)

## Electrolyzer ##

#Constants

F_C = 96485 #Faraday constant [C/mol]

#Molecular Masses

M_H2O = 18.01528 #[g/mol]

M_H2 = 2.016 #[g/mol]

M_O2 = 31.999 #[g/mol]

#Operational Conditions

T_Elr = 353 #[K]

P_ELr = 100 #[kPa]

#Structural Parameters

D_Elit_1 = 50 * 10**(-6) #Electrode thickness [m] #Nafion 212

lambda_a1 = 14

lambda_c1 = 10

#Partial Pressures

P0_P_H2 = P_ELr * 0.01

P0_P_O2 = P_ELr * 0.01

P0_P_H2O = P_ELr * 0.01

#Current Parameters

#Exchange Current Density

alfa_0_cath = 0.5

alfa_0_anod = 0.5

J_0_cath = 10 #[A/m^2]

J_0_anod = 1.0 * 10**(-5) #[A/m^2]

#Operating current density

#Creating a sequence of numbers to determine the power required for the electrolyzer at different current densities

J_1 = np.linspace(1, 20000, num=2000) #5000 #[A/m^2]

J_1 = np.array(J_1)

#Standard Potential

E_P0 = standard_potential(T_Elr)

#print(E_P0)

#Reversible Potential

E_V = reversible_potential(E_P0, R_GC, T_Elr, F_C, P0_P_H2, P0_P_O2, P0_P_H2O)

#print(E_V)

#Ohmic Overpotential

eta_ohmic = ohmic_overpot(lambda_a1, lambda_c1, D_Elit_1, T_Elr, J_1)

#print(eta_ohmic)

#Activation Overpotential

eta_act_cath = activation_overpot(R_GC, T_Elr, F_C, J_1, J_0_cath, alfa_0_cath)

eta_act_anod = activation_overpot(R_GC, T_Elr, F_C, J_1, J_0_anod, alfa_0_anod)

#print(eta_act_cath, eta_act_anod)

#Actual potential

V_R = E_V + eta_act_cath + eta_act_anod + eta_ohmic

#print(V_R)

#Power density

W_flux_electrolyzer = V_R * J_1 / 1000

#print(W_flux_electrolyzer)

## Connecting wind turbine and electrolyzer to each other

#Creating a relationship between the current density and the power density of the electrolyzer using linear regression

LinReg = np.polyfit(J_1, W_flux_electrolyzer, 1)

#print(LinReg)

W_A = W_flux_electrolyzer

W_P = LinReg[0] * J_1 + LinReg[1]

# Errors

MSE = sum((W_A - W_P)**2)/len(W_A)

#print('RMSE =', np.sqrt(MSE))

TSS = sum((W_A - np.mean(W_A))**2)

R2 = 1 - (len(W_A)*MSE/TSS)

#print('R2 =', R2)

#The current density created for the electrolyzer based on the output power from the wind turbine

A_Elcr = 2.25 #The active surface of the electrolyzer [m2]

#Stack Parameters

A_Cell = 0.0625 #[m^2] 25*25 [cm] >>>

n_Cell = A_Elcr / A_Cell

#print(n_Cell) # 36

J_WindTur = (TurPow/A_Elcr - LinReg[1])/LinReg[0]

J_WindTurDF = pd.DataFrame(J_WindTur)

#print(J_WindTurDF.describe()) # max = 1.965922e+04 >>> J_WindTur < 20000 #[A/m^2]

#print(sum(J_WindTur)/31557600)

#Calculation of hydrogen produced based on the density of the current entered into the electrolyzer

m_dot_H2_Prod = (0.5 * J_WindTur * M_H2 * A_Elcr) / (F_C * 1000)

Mass_H2_Ann = sum(m_dot_H2_Prod) #For total annual production equal to 1825 [kg] = 5 [kg/day]

m_dot_H2_Prod_Ave = Mass_H2_Ann / 31557600

#print(Mass_H2_Ann, m_dot_H2_Prod_Ave) # 1825.3393376589743 [kg] 5.784151322213902e-05 [kg/s]

## Electrolyzer calculations with current densities obtained from wind turbine power

J_New = J_WindTur

#Ohmic Overpotential

eta_ohmic = ohmic_overpot(lambda_a1, lambda_c1, D_Elit_1, T_Elr, J_New)

eta_ohmicDF = pd.DataFrame(eta_ohmic)

#print(eta_ohmicDF.describe())

#print(sum(eta_ohmic)/31557600)

#Activation Overpotential

eta_act_cath = activation_overpot(R_GC, T_Elr, F_C, J_New, J_0_cath, alfa_0_cath)

eta_act_anod = activation_overpot(R_GC, T_Elr, F_C, J_New, J_0_anod, alfa_0_anod)

eta_act_cathDF = pd.DataFrame(eta_act_cath)

eta_act_anodDF = pd.DataFrame(eta_act_anod)

#print(eta_act_cathDF.describe())

#print(eta_act_anodDF.describe())

#print(sum(eta_act_cath)/31557600)

#print(sum(eta_act_anod)/31557600)

#Actual potential

V_R = E_V + eta_act_cath + eta_act_anod + eta_ohmic

V_RDF = pd.DataFrame(V_R)

#print(V_RDF.describe())

#print(sum(V_R)/31557600)

#Mass Transfer Rates

N_H2O_C = 0.5 * J_New / F_C

N_H2_P = 0.5 * J_New / F_C

N_O2_P = 0.25 * J_New / F_C

#print(sum(N_H2O_C)/31557600)

#print(sum(N_H2_P)/31557600)

#print(sum(N_O2_P)/31557600)

#Power density

W_flux_electrolyzer = V_R * J_New / 1000

W_flux_electrolyzerDF = pd.DataFrame(W_flux_electrolyzer)

#print(W_flux_electrolyzerDF.describe())

#print(sum(W_flux_electrolyzer)/31557600)

#Entropy Generation

S_gen_electrolyzer = 2 * F_C * (V_R - E_V) / (1000 * T_Elr) #[kJ/mol-K] @ Mole of H2 produced

S_gen_electrolyzerDF = pd.DataFrame(S_gen_electrolyzer)

#print(S_gen_electrolyzerDF.describe())

#print(sum(S_gen_electrolyzer)/31557600)

#Heat density

#Entropy of Material

Entropy_H2O = 1.07 * M_H2O / 1000 # @ T_Elr = 353 #[K] & P_ELr = 100 #[kPa]

Entropy_H2 = 55.86 * M_H2 / 1000 # @ T_Elr = 353 #[K] & P_ELr = 100 #[kPa]

Entropy_O2 = 6.567 * M_O2 / 1000 # @ T_Elr = 353 #[K] & P_ELr = 100 #[kPa]

Q_flux_electrolyzer = 0.5 * J_New / F_C * (T_Elr * (((Entropy_H2 + 0.5 * Entropy_O2) - Entropy_H2O) - S_gen_electrolyzer)) # [kW/m^2]

Q_flux_electrolyzerDF = pd.DataFrame(Q_flux_electrolyzer)

#print(Q_flux_electrolyzerDF.describe()) # All values are negative >>> Heat is not required

#print(sum(Q_flux_electrolyzer)/31557600)

#Heat Transfer Rate #### Elc

Q_dot_electrolyzer = Q_flux_electrolyzer * A_Elcr

Q_dot_electrolyzerAve = sum(Q_dot_electrolyzer)/31557600 # 31,557,600 [s] = 1 [year]

#print(Q_dot_electrolyzerAve)

#Work Transfer Rate

W_dot_electrolyzer_req = W_flux_electrolyzer * A_Elcr

W_dot_electrolyzer_reqAve = sum(W_dot_electrolyzer_req)/31557600

#print(W_dot_electrolyzer_reqAve, sum(W_dot_electrolyzer_req))

W_dot_electrolyzer_reqDF = pd.DataFrame(W_dot_electrolyzer_req)

#print(W_dot_electrolyzer_reqDF.describe())

#Entropy Rate duo to Heat

S_dot_Heat_electrolyzer = Q_dot_electrolyzer / T_Elr

S_dot_Heat_electrolyzerAve = sum(S_dot_Heat_electrolyzer)/31557600

#print(S_dot_Heat_electrolyzerAve)

#Entropy Generation Rate

MF_H2_req = m_dot_H2_Prod * 1000 / M_H2

#print(sum(MF_H2_req)/31557600)

S_dot_gen_electrolyzer = S_gen_electrolyzer * MF_H2_req #[kW/K] @ Molar Flowrate H2

S_dot_gen_electrolyzerAve = sum(S_dot_gen_electrolyzer)/31557600

#print(S_dot_gen_electrolyzerAve)

#Exergy Rate duo to Heat

Ex_dot_Heat_electrolyzer = Q_dot_electrolyzer * (1 - T_env/T_Elr)

Ex_dot_Heat_electrolyzerAve = sum(Ex_dot_Heat_electrolyzer)/31557600

#print(Ex_dot_Heat_electrolyzerAve)

#Exergy Destruction Rate

Ex_dot_Des_electrolyzer = T_env * S_dot_gen_electrolyzer

Ex_dot_Des_electrolyzerAve = sum(Ex_dot_Des_electrolyzer)/31557600

#print(Ex_dot_Des_electrolyzerAve)

# Streams of Electrolyzer

#Input Stream:

m_dot_H2O_R = MF_H2_req * M_H2O / 1000

m_dot_H2O_R_Ave = sum(m_dot_H2O_R)/31557600

h_In_H2O = 335.3 #[kJ/k] ### S.EW

s_In_H2O = 1.07 #[kJ/kg-K]

h_In_H2O_env = 112.65 #[kJ/kg] ### S.W

s_In_H2O_env = 0.39306 #[kJ/kg-K]

ex_In_H2O = exergy(h_In_H2O, h_In_H2O_env, s_In_H2O, s_In_H2O_env, T_env)

#print(m_dot_H2O_R_Ave, ex_In_H2O)

#Output Streams:

#1. O2 Stream

m_dot_O2_P = 0.5 * MF_H2_req * M_O2 / 1000

m_dot_O2_P_Ave = sum(m_dot_O2_P)/31557600

h_Out_O2 = 321.8 #[kJ/kg] ### S1.O

s_Out_O2 = 6.567 #[kJ/kg-K]

h_Out_O2_env = 272.71 #[kJ/kg] ### S2.O

s_Out_O2_env = 6.4163 #[kJ/kg-K]

ex_Out_O2 = exergy(h_Out_O2, h_Out_O2_env, s_Out_O2, s_Out_O2_env, T_env)

#print(m_dot_O2_P_Ave, ex_Out_O2)

#2. H2 Stream

m_dot_H2_P = m_dot_H2_Prod

m_dot_H2_P_Ave = sum(m_dot_H2_P)/31557600

h_Out_H2 = 4722 #[kJ/kg] ### S1.H

s_Out_H2 = 55.86 #[kJ/kg-K]

h_Out_H2_env = 3958.3 #[kJ/kg] ### S2.H

s_Out_H2_env = 53.519 #[kJ/kg-K]

ex_Out_H2 = exergy(h_Out_H2, h_Out_H2_env, s_Out_H2, s_Out_H2_env, T_env)

#print(m_dot_H2_P_Ave,ex_Out_H2)

#Saturated Steam X = 1 @ T = 372.76 [K] ### S.SW

h_gas = 2674.9 #[kJ/kg]

s_gas = 7.3588 #[kJ/kg-K]

ex_gas = exergy(h_gas, h_In_H2O_env, s_gas, s_In_H2O_env, T_env)

#print(ex_gas)

Delta_H_H2O_R = m_dot_H2O_R * (h_In_H2O - h_In_H2O_env)

Delta_H_O2_P = m_dot_O2_P * (h_Out_O2 - h_Out_O2_env)

Delta_H_H2_P = m_dot_H2_P * (h_Out_H2 - h_Out_H2_env)

Delta_H_H2O_V = m_dot_H2O_R * (h_gas - h_In_H2O)

h_H2O_In_Hex = h_gas - (Delta_H_H2O_V + Delta_H_O2_P + Delta_H_H2_P) / m_dot_H2O_R ### S.WW

#print(h_H2O_In_Hex[0]) #206.24096483651647 [kJ/kg] >> T in Hex = 322.2 [K] >>>

s_H2O_In_Hex = 0.692 #[kJ/kg-K]

ex_H2O_In_Hex = exergy(h_H2O_In_Hex[0], h_In_H2O_env, s_H2O_In_Hex, s_In_H2O_env, T_env)

#print(ex_H2O_In_Hex)

#for Electric Heater #### EWH

C_P_Heater = 0.95 # Electric Heater efficiency

W_dot_EH = m_dot_H2O_R * (h_H2O_In_Hex[0] - h_In_H2O_env) * 1.1 / C_P_Heater # 1.1 for brine water

W_dot_EH_total = sum(W_dot_EH)

#print(W_dot_EH_total)

W_dot_EH_Ave = W_dot_EH_total/31557600

#print(W_dot_EH_Ave)

# @ start of process:

W_dot_EH_S = m_dot_H2O_R * (h_gas - h_In_H2O_env) * 1.1 / C_P_Heater # 1.1 for brine water

W_dot_EH_total_S = sum(W_dot_EH_S)

#print(W_dot_EH_total_S)

W_dot_EH_Ave_S = W_dot_EH_total_S/31557600

#print(W_dot_EH_Ave_S)

#for Hex #### Hex

S_dot_gen_Hex = m_dot_H2O_R * (s_In_H2O - s_H2O_In_Hex - s_gas) + m_dot_H2_P * (s_Out_H2_env - s_Out_H2 ) + m_dot_O2_P * (s_Out_O2_env - s_Out_O2)

Ex_dot_Des_Hex = S_dot_gen_Hex * T_env

S_dot_gen_Hex_Ave = sum(S_dot_gen_Hex)/31557600

Ex_dot_Des_Hex_Ave = sum(Ex_dot_Des_Hex)/31557600

#print(S_dot_gen_Hex_Ave, Ex_dot_Des_Hex_Ave)

## Energy and Exergy Efficiencies ##

LHV_H2 = 119930 #[kJ/kg] @ T0 = 25 [ºC] and P0 = 1 [atm] Standard Conditions

ex_H2 = 116760 #[kJ/kg] @ T0 = 298 [K] and P0 = 1.013 [bar] Standard Conditions

# @ start of process:

eta_en_Elc = (m_dot_H2_P * LHV_H2) / (W_dot_electrolyzer_req + W_dot_EH_S)

psi_ex_Elc = (m_dot_H2_P * ex_H2) / (W_dot_electrolyzer_req + W_dot_EH_S)

#print(np.mean(eta_en_Elc), np.mean(psi_ex_Elc))

# @ start of process = 0.5687611915723935 0.5537276471941376

# @ steady-state:

eta_en_Elc = (m_dot_H2_P * LHV_H2) / (W_dot_electrolyzer_req + W_dot_EH)

psi_ex_Elc = (m_dot_H2_P * ex_H2) / (W_dot_electrolyzer_req + W_dot_EH)

#print(np.mean(eta_en_Elc), np.mean(psi_ex_Elc))

# @ steady-state = 0.6472563251538656 0.630147990702623

#Final calculation of wind turbine surface

PowerTotal = W_dot_electrolyzer_req + W_dot_EH #### VAWT

PowerTotal_Ave = sum(PowerTotal)/31557600

#print(PowerTotal_Ave)

A_tur = final_surface(PowerTotal, C_P1, rho_Air1, V_air)

A_turDF = pd.DataFrame(A_tur)

A_turDF.replace([np.inf, -np.inf], 0, inplace=True)

#A_turDF.replace([np.inf, -np.inf], np.nan, inplace=True)

#A_turDF.dropna(axis=0)

#print(A_turDF.describe())

Final_Surface = np.mean(A_tur)

#print(Final_Surface)

TurPowF = turbine_power(C_P1, rho_Air1, Final_Surface, V_air)

TurPowFDF = pd.DataFrame(TurPowF)

#print(TurPowFDF.describe()) #[kW] >> rated power = 8.934470e+01 [kW] ~ 90 [kW]

#print('Sum of the annual power output of Wind Turbine:', sum(TurPow)) # >> 338980715.9493483 [kW-year]

#print(sum(TurPow)/31557600)

#Calculation of wind turbine dimensions

R_HtoR = 2.8 #Height-to-Radius ratio >> Height = Radius * 2.8, Height = A1 / 2*Radius >>>

Radius = sqrt(Final_Surface / (2.8*2))

Height = Radius * 2.8

#print(Radius, Height) #4.755597222931282 13.315672224207589[m]

#Overall mini-Factory efficiency

eta_en_Total = (m_dot_H2_P * LHV_H2) / TurPowI

psi_ex_Total = (m_dot_H2_P * ex_H2) / TurPowI

#print(np.mean(eta_en_Total), np.mean(psi_ex_Total))

# eta_en_Total_Ave = 0.27762240732253884

# psi_ex_Total_Ave = 0.27028426814791684

#Calculation of appropriate height for winds less than the calculated wind

#Hellmann exponent

a_H = 0.34 # Neutral air above human inhabited areas

V10 = 6 # 1 , 2, 3, 4, 5, 6 velocity of the wind [m/s], at height 10 meters

V_Final = Wind_Speed

H_Final = height(Wind_Speed, V10, a_H)

#print(H_Final) # [m] 3059.0210861373844, 398.29066752980486, 120.86057681916998, 51.85824202396918, 26.902233646610764, 15.736288983914035
